# STAMBP promotes lung adenocarcinoma metastasis by regulating the EGFR/MAPK signaling pathway

**DOI:** 10.1016/j.neo.2021.05.011

**Published:** 2021-06-05

**Authors:** Hui Xu, Xiaomei Yang, Xiaofeng Xuan, Di Wu, Jieru Zhang, Xinchun Xu, Yuanjie Zhao, Chunping Ma, Dawei Li

**Affiliations:** a Department of Thoracic Surgery, The Affiliated Zhangjiagang Hospital of Soochow University, 68 Jiyang West Road, Suzhou, 215600, China; b Center for Translational Medicine, The Affiliated Zhangjiagang Hospital of Soochow University, 68 Jiyang West Road, Suzhou, 215600, China; c Department of Emergency, The Affiliated Zhangjiagang Hospital of Soochow University, 68 Jiyang West Road, Suzhou, 215600, China; d Department of Respiratory & Critical Care Medicine, The Affiliated Zhangjiagang Hospital of Soochow University, 68 Jiyang West Road, Suzhou, 215600, China; e Department of Ultrasound, The Affiliated Zhangjiagang Hospital of Soochow University, 68 Jiyang West Road, Suzhou, 215600, China; f Department of General Surgery, The Affiliated Zhangjiagang Hospital of Soochow University, 68 Jiyang West Road, Suzhou, 215600, China; g Lead Contact

**Keywords:** Lung adenocarcinoma, STAMBP, Metastasis, EGFR, MAPK, ERK

## Abstract

Tumor metastasis is a leading cause of death in lung adenocarcinoma (LUAD) patients, but the molecular events that regulate metastasis have not been completely elucidated. STAMBP is a deubiquitinating enzyme of the Jab1/MPN metalloenzyme family that regulates the stability of substrates in cells by specifically removing ubiquitin molecules. We found that STAMBP expression was increased in the cytoplasm of tumor cells from LUAD patients. The STAMBP level was closely associated with tumor size, lymph node invasion and neoplasm disease stage. A high STAMBP level predicted poor overall survival and disease-free survival in LUAD patients. STAMBP overexpression promoted cell migration and invasion, whereas STAMBP knockdown attenuated these processes in LUAD cells after epidermal growth factor treatment. Mechanistically, increased STAMBP expression promoted the stabilization of Epidermal growth factor receptor (EGFR), whereas STAMBP knockdown induced the degradation of EGFR. STAMBP may deubiquitinate EGFR by localizing in early endosomes and increase EGFR membrane localization in LUAD cells. The overexpression of STAMBP triggered the activation of MAPK signaling after epidermal growth factor treatment. In contrast, this activation was attenuated in STAMBP knockdown cells. Small molecule inhibitors of EGFR and MAPK signaling pathway may block STAMBP-induced cell mobility and invasion as well as ERK activation in cells. Importantly, STAMBP knockdown suppressed LUAD tumor growth and metastasis by regulating the EGFR-mediated ERK activation in a xenograft mouse model. Our findings identified STAMBP as a novel potential target for LUAD therapy.

## Introduction

Lung cancer is the leading cause of cancer-related mortality worldwide, with an overall 5-y survival rate of 19% [1]. Non-small cell lung carcinoma (NSCLC) accounts for approximately 85% of all lung cancers and includes squamous cell carcinoma (LUSC), adenocarcinoma (LUAD) and large cell carcinoma [2]. The current treatments for NSCLC include surgery, radiation, chemotherapy and targeted therapies. Despite advances in treatment options, prognosis remains poor because of the presence of metastatic tumors in more than half of NSCLC patients at the time of diagnosis [1,3]. The molecular events that regulate NSCLC metastasis have not been completely elucidated.

Epidermal growth factor receptor (EGFR), a receptor tyrosine kinase of the HER/erbB family, plays key roles in essential cellular functions, including proliferation and migration [[4], [5], [6]]. Upon binding to its ligand, EGFR dimerizes and is autophosphorylated to trigger downstream intracellular signaling via RAS/RAF/MEK/ERK phosphorylation. EGFR pathway activation ultimately results in increased proliferation, angiogenesis, and metastasis and decreased apoptosis [4−6]. The duration and strength of EGFR signaling is tightly controlled via ubiquitin-dependent endocytosis and lysosomal degradation [7,8]. Although EGFR is overexpressed in most NSCLC patients [9], the molecular events leading to elevated EGFR levels remain unknown. Insight into the molecular mechanism by which EGFR stability is regulated will contribute to the development of alternative potential drug targets.

STAMBP, a member of the Jab1/MPN metalloenzyme family of deubiquitinating enzymes (DUBs), specifically cleaves K63-linked polyubiquitination chains from substrates [[10], [11], [12]]. STAMBP is implicated in the endosome-lysosome pathway and shuttles ubiquitinated substrate proteins to lysosomes for degradation [[10], [11], [12], [13], [14]]. K63-linked mono- or polyubiquitination can trigger substrate protein endocytosis and recruitment of the endosomal sorting complexes required for transport (ESCRT) machinery [15]. STAMBP regulates protein degradation by binding to the ESCRT-0 protein STAM and the charged multivesicular body proteins of the ESCRT-III complex [10,[16], [17], [18], [19], [20]]. STAMBP knockout mice exhibit postnatal growth delays and die at an early stage after birth due to cell apoptosis and loss of neurons in the hippocampus [21]. STAMBP mutation is observed in microcephaly capillary malformation syndrome and is considered a main cause of the disease [[22], [23], [24], [25]]. STAMBP is implicated in the inflammatory response by promoting the deubiquitination and stabilization of NALP7 and increasing the activity of inflammasomes [26]. STAMBP has been shown to deubiquitinate EGFR and therefore promote the recycling of the receptor [8,10]. However, STAMBP may also induce the degradation of cell membrane receptors and gap junctions [16,[27], [28], [29], [30]]. Accumulating evidence has revealed that STAMBP, as an ESCRT-III-associated enzyme, contributes to EGFR degradation by deubiquitinating the cargo in MVB/late endosomes [19,30,31]. Therefore, the effect of STAMBP on EGFR stability remains controversial and needs to be further elucidated. Although STAMBP regulates melanoma metastasis through SLUG stabilization [32], its roles in NSCLC progression remain completely unknown.

Here, we assessed STAMBP expression and its clinical correlation in NSCLC patients. Furthermore, we investigated the effects of STAMBP on cell mobility and invasion in vitro and tumor metastasis in a xenograft mouse model in vivo. We found that targeting STAMBP suppresses LUAD metastasis by regulating EGFR stability and MAPK activation. We propose that STAMBP may be a novel promising target for LUAD therapy.

## Materials and methods

### Patients

We evaluated the expression of STAMBP in 24 NSCLC and matched non-cancerous tissues from NSCLC patients. These patients were diagnosed as NSCLC by pathology and underwent lung cancer surgery in Zhangjiagang Hospital Affiliated to Suzhou University from March 2019 to February 2020. None of these patients received adjuvant chemotherapy or radiotherapy before operation. According to the guidelines from American Joint Committee on Cancer (http://www.cancerstaging.org/), the tumor staging at the time of diagnosis was evaluated. The tumor samples were carefully isolated during surgery and the matched noncancerous lung tissues were collected at least one centimeter away from the edge of the lesion. The tissue samples were immediately frozen at -80˚C for protein extraction. Written permission was requested and received from these NSCLC patients in the study. The use of human specimens has been approved by Zhangjiagang Hospital Institutional Review Committee (No. 2019001). To further determine STAMBP expression in LUAD tissues, we purchased human LUAD tissue microarray (#HLugA150CS03, Shanghai Outdo Biotech Company, Shanghai, China). Seventy-five paired tissues from tumor and adjacent non-cancerous tissues were also assessed by immunohistochemistry (IHC). The demographic and clinical features of the patients are shown in Supplementary Table 1 and 2.

We used the publicly available TCGA databases to assess the mRNA expression of STAMBP in tissues from normal lung and NSCLC patients. The mRNA expression of STAMBP was retrieved and analyzed from 59 normal lung tissues and 515 LUAD tissues and 52 normal lung tissues and 503 LUSC tissues (http://ualcan.path.uab.edu/). The mRNA level was also assessed from 10 paired LUAD and adjacent non-cancerous tissues from Gene Expression Omnibus (GEO; https://www.ncbi.nlm.nih.gov/geoprofiles/17277280) [33]. We also used the publicly available clinical information provided by cBioportal for Cancer Genomics (TCGA) databases (http://www.cbioportal.org/) [34,35] to assess the correlation between STAMBP and clinical pathological status as well as patient survival in NSCLC patients. We retrieved clinical information from NSCLC patients including 496 LUAD and 484 LUSC patients for correlation analysis. We stratified 485 LUAD and 478 LUSC patients into higher and lower STAMBP expression levels for the overall survival (OS) analyses. We extracted data from 209 LUAD patients with higher and lower quartiles for STAMBP expression for the disease-free survival (DFS) analyses. We stratified 364 LUSC patients into higher and lower STAMBP expression levels for the DFS analyses.

### Cell culture and stable lines

The human LUAD cell lines H1299 and A549 were kindly provided by the Stem Cell Bank, Chinese Academy of Sciences and were proven to be negative for mycoplasma contamination. The cells were cultured in Dulbecco's modified Eagle's medium (DMEM, Corning, AZ, USA) supplemented with 10% fetal bovine serum (FBS) and antibiotics (100 U/ml penicillin and 100 µg/ml streptomycin) in a humidified incubator at 37°C in 5% CO_2_. To obtain stable cells expressing FLAG- and HA-tagged STAMBP (FH-STAMBP), H1299 cells were transfected with pCIN4-FH-STAMBP expression constructs and selected for 2 wk with 1 mg/ml G418 (Gibco, NY, USA). To generate stable knockdown A549 cells, the cells were infected with lentivirus expressing control or STAMBP shRNA at a multiplicity of infection of 60 in a solution containing 5 μg/ml polybrene. Cells at a confluence of 20-30% were selected with 1 μg/ml puromycin dihydrochloride (Beyotime, Shanghai, China) for 14 d.

### Cell transfection and drug treatment

The cells were transfected with specific siRNAs or plasmids using Lipofectamine 2000 (Invitrogen, CA, USA) according to the manufacturer's protocol. For EGF treatment, the cells were starved in medium containing 0.1% serum for 24 h, and then, 100 ng/ml EGF was added to the culture. In some cases, the specific inhibitors AG1478, salirasib, TAK580, LY3009120 and LY3214996 from MedChem Express (NJ, USA) and U0126 and LY294002 from Cell Signaling Technology (CST, MA, USA) were used to treat the cells for the indicated times. BC-1471 was purchased from AOBIOUS (Cat No: AOB36470, MA, USA).

### Western blotting and antibodies

The tissues and cells were collected and homogenized in FLAG lysis buffer as previously described [36]. After electrophoresis, the proteins were transferred onto nitrocellulose membranes (GE Healthcare, WI, USA). The membranes were blocked with 5% nonfat milk for 1 h at room temperature and incubated overnight at 4°C with primary antibodies. After 3 washes, the membranes were incubated with the appropriate secondary antibodies for 1 h at RT. The signals were developed using an enhanced chemiluminescence (Millipore, MA, USA) and ChemiDoc XRS (Bio-Rad, CA, USA) detection system. STAMBP (sc-271641), EGFR (sc-373746), and STAM (sc-133093) antibodies were purchased from Santa Cruz Biotechnology (CA, USA). EGFR (4267S), RAB5 (3547T), RAB7 (9367T), P-EGFR (3777T), MEK (9126S), P-MEK (9121S), ERK (9101S) and Phospho-p44/42 MAPK (Also known as ERK1/2, Thr202/Tyr204, 9102S) antibodies were purchased from Cell Signaling Technology (CST, MA, USA). β-Actin (A3853) and ubiquitin (FK2, ST1200) primary antibodies were purchased from Sigma-Aldrich (MS, USA). The peroxidase-linked secondary antibodies (NA931 and NA935) were purchased from GE healthcare. Alexa Fluor 488 donkey anti-rabbit and Alexa Fluor 555 donkey anti-mouse secondary antibodies were purchased from Invitrogen (CA, USA).

### Immunohistochemistry (IHC)

The tissues were formalin-fixed and paraffin-embedded. IHC staining was performed using the peroxidase technique, as previously described [37]. Briefly, the sections were cut into 4 µm slices, deparaffinized and rehydrated. Epitope retrieval was performed in EDTA buffer (pH 9.0) in a water bath at 95°C for 20 min with cooling for 10 min before immunostaining. The slides were further treated with 6% H_2_O_2_ for 10 min to block the endogenous peroxidase activity. The tissues were incubated with STAMBP (1:25 dilution), EGFR (4267S, CST, 1:2500) and p-ERK (9101S, CST, 1:5000) primary antibodies overnight at 4°C after blocking and then exposed to an EnVision FLEX mouse or rabbit HRP-conjugated secondary antibody solution (DAKO, Denmark). Finally, the slides were incubated with a 3, 3’-diaminobenzidine (DAB) solution (DAKO) for 1 to 5 min and counterstained with hematoxylin for 2 min. The digitized images were acquired using Aperio ImageScope software (Aperio XT, Leica Microsystems). Nucleic and cytoplasmic scoring was evaluated by 2 investigators in a blinded manner, as previously described [36].

### RNA interference

H1299 and A549 cells were transiently transfected with specific siRNAs using Lipofectamine 2000 (Invitrogen, USA) according to manufacturer's protocol. Two individual STAMBP siRNAs (STAMBP siRNA-1 and STAMBP siRNA-2) and scrambled negative control siRNA (NC) were synthesized by Genepharma (Shanghai, China). The nucleotide sequences of siRNAs were as follows: STAMBP siRNA-1: sense, 5’-CAUCCUCUAUAACAAGUAUdTdT-3’, antisense, 5’-AUACUUGUUAUAGAGGAUGdTdT-3’; STAMBP siRNA-2: sense, 5’-GAGUUGAGAUUAUCCGAAdTdT-3’, antisense, 5’-AUUCGGAUAAUCUCAACUCdTdT-3’; and NC: sense, 5’-UUCUCCGAACGUGUCACGUTT-3’, antisense, 5’-ACGUGACACGUUCGGAGAATT-3’.

We used lentivirus mediated shRNA expression to stably knock down STAMBP expression in A549 cells. The control and STAMBP shRNA sequences were cloned into lentivirus vector LV2 (U6/Puro) as follows (targeted sequence is italic and bold): Control shRNA sequence, 5’-TGTTCTCCGAACGTGTCACGTTTCAAGAGAACGTGACACGTTCGGAGAACTTTTTTC-3′; STAMBP shRNA sequence, 5’-TGCATCCTCTATAACAAGTATTTCAAGAGAATACTTGTTATAGAGGATGCTTTTTTC-3’. The lentivirus constructs were co-transfected with helper vectors, pGag/Pol, pRev and pVSV-G into 293T packaging cells. The lentivirus particles were purified by ultra-centrifuge process after the cell supernatants were collected after 72 h.

### Real-time PCR (RT-PCR)

Total cellular RNA was isolated using Trizol reagent (Invitrogen, CA, USA) following the manufacturer's protocol. RNA was reverse-transcribed into cDNA using the Thermo Scientific RevertAid H Minus First Strand cDNA Synthesis Kit (Thermofisher, MA, USA). Real-time PCR (RT-PCR) was performed in triplicate using SYBR green mix (Applied Biosystems, Foster City, CA, USA) and a QuantStudio Dx Real-Time PCR Instrument (Applied Biosystems) under the following conditions: 10 min at 95 °C followed by 40 cycles of 95 °C for 15 s and 60°C for 1 min. β-Actin was used as an internal reference for normalization. The sequences of primers (Sangon, Shanghai, China) were as follows: STAMBP, F: 5′-TCCACACAGCAAGGATCCAC-3′, R: 5′-GGTCACTGCTCTGTCCACAA-3′, EGFR, F: 5’- GCGTCTCTTGCCGGAATGT-3’, R: 5’- GGCTCACCCTCCAGAAGGTT-3’, β-Actin, F: 5’-AGAGCTACGAGCTGCCTGAC-3’, R: 5’-AGCACTGTGTTGGCGTACAG-3’.

### In vitro and in vivo deubiquitination assays

EGFR protein complex was purified in non-denaturing conditions using BC100 lysis buffer without EDTA (20 mM Tris-HCl, pH 7.9, 100 mM NaCl, 10% glycerol, 0.2% Triton X-100, and freshly supplemented protease inhibitor) from H1299 cells. EGFR protein complex was immunoprecipitated by EGFR antibody and protein G agarose (Santa Cruz Biotechnology) overnight at 4°C. FH-STAMBP and FH-STAMBP (D348A; catalytically inactive mutants) constructs were transfected into 293T cells. The proteins were purified from 293T cells using BC300 high salt lysis buffer without EDTA (20 mM Tris-HCl, pH 7.9, 300 mM NaCl, 10% glycerol, 0.2% Triton X-100, and freshly supplemented protease inhibitor). Anti-FLAG antibody conjugated M2 beads (FLAG/M2, Sigma, USA) were used to immunoprecipitate STAMBP proteins. FLAG peptides (Bimake.com) were used to elute the purified proteins. In vitro deubiquitination assay was performed by incubating EGFR complex with FH-STAMBP or FH-STAMBP (D348A) in a deubiquitination buffer (50mM Tris-HCl pH 8.0, 50mM NaCl, 10mM DTT, 5% glycerol) for 120 min at 37°C. The reaction mixture was subsequently resolved on 8% SDS-PAGE for Western blot.

In vivo deubiquitination assay was performed using STABMP overexpression or knockdown cells without or with EGF treatment. The cells were lysed in cold BC100 lysis buffer without EDTA. Total EGFR protein was immunoprecipitated from cell extracts by EGFR antibody and protein G agarose overnight at 4°C. After extensive washing, the elution was resolved by SDS-PAGE and detected with anti-ubiquitin antibody.

### Immunofluorescence staining

H1299 and H-1 cells were plated into slides in 24-well plates and grown to 40% confluence in DMEM containing 10% FBS. The cells were not treated or treated with 100 ng/ml EGF for 20 min, and then fixed in 4% paraformaldehyde for 15 min. After 3 washes, the cells were permeabilized with 0.3% Triton X-100 in phosphate-buffered saline, blocked with 10% goat serum for at least 1h at room temperature and incubated at 4°C overnight with the following primary antibodies at a dilution of 1:200: rabbit anti-EGFR, rabbit anti-RAB5, rabbit anti-RAB7 and mouse anti-STAMBP antibodies. After extensive washing, the sections were incubated with Alexa Fluor 488 donkey anti-rabbit and Alexa Fluor 555 donkey anti-mouse secondary antibodies at a dilution of 1:3000 for 1 h at room temperature. Finally, the slides were counterstained with 4’, 6-diamidino-2-phenylindole dihydrochloride for 10 min and were observed with a laser confocal microscope Leica DMi8 (Leica Microsystems, Germany). The images were obtained using LAS X software.

### Cell viability, wound closure and transwell motility assays

Cell counting kit-8 (Dojindo, Kumamoto, Japan) was used to measure cell growth after stimulation with or without EGF (100 ng/ml) for indicated times. Parental H1299 and the stable cell lines were seeded at a density of 500 cells each well in 96-well plates. Cell growth was monitored at 1, 3, 5 and 7 d. H1299 or A549 cells were seeded at a density of 1000 cells each well in 96-well plates before the cells were transfected with siRNAs. Cell viability was monitored at 1, 3 and 5 d after cells were transfected with control and STAMBP siRNAs. A microplate reader (Thermo Scientific) was used to measure absorbance of each well at 450 nm. The results are shown as the mean optical density (OD) ± SD.

### Wound closure and transwell motility assays

H1299 and A549 cells were plated into 6-well plates and grown to 80% confluence in DMEM containing 10% FBS. The medium was changed into DMEM without FBS for 24 h. The wound was induced by manually scraping the cell monolayer with a pipette tip with or without the EGF (100 ng/ml) added into medium. The medium was changed every 24 h with a new EGF added. The wound closure was monitored by microscopy for indicated times. Transwell motility assays were performed utilizing 24-well plates, 8.0µm pore size PET track-etched membrane transwell filters (BD falcon, NJ, USA). Single cell suspensions were seeded in clear media, containing 0.1% of FBS onto the upper surface of the filter and allowed to migrate toward the bottom of the transwell which contained 10% FBS. For these assays, 5 × 10^3^ stable cells or 2 × 10^4^ siRNA treated cells were stimulated with EGF for 48 or 72 h. Then the cells that migrated into the underside of the filter were fixed, stained with 0.5% crystal violet, and counted by microscopy at × 50 and 100 in 3 random fields. For cell invasion assay, the matrigel matrix (Corning, Glendale, AZ, USA) was put in the upper surface of the filter before the medium containing cells were added.

### Xenograft tumor growth and metastasis in nude mice

A total of 24 6-wk-old female nude mice (NU/NU nude mice) were purchased from Charles River Laboratories and kept in individual ventilation cages on exhaust-ventilated closed-system cage racks in animal house. All mice were housed in a temperature-controlled room (22 ± 2°C) with 40% to 60% humidity and a light/dark cycle of 12 h/12 h. All animal experiments were approved by the Animal Ethics Committee of Soochow University, China. 24 mice were randomly divided into 2 groups with 12 mice for each group. A total of 5 × 10^6^ A549 cells stably expressing control shRNA or STAMBP shRNA were suspended in 50 µL phosphate-buffered saline and 50 µL matrigel matrix and injected into left lung of each mouse after exposure of pleura by surgery. The mice were anaesthetized using sevoflurane inhalation before and during surgery. Two mice died from over anesthesia in each group. The mice were euthanized 6 wk after tumor implantation. The lung tumor was dissected and tumor volume was calculated as previously described [37] by the formula V =π × L × W^2^/6, where L represents the longest dimension and W the shortest dimension of the tumor. The number of metastasis lesions on the surface of chest wall was counted before pathological analysis.

### Statistical analysis

Statistical analysis was performed using GraphPad Instat software 5.01 (GraphPad Software Inc., CA, USA). The quantitative data are presented as the mean values ± standard deviations. The results were calculated using Student's t test for comparison of 2 groups and one-way ANOVA for comparisons of multiple groups. Chi-squared or Fisher's exact tests were used to analyze the correlation between STAMBP expression and demographic and clinical features. Survival curves were plotted using the Kaplan-Meier method and compared with the log-rank test or Gehan-Breslow-Wilcoxon test. Statistical significance was accepted at *P* < 0.05.

## Results

### STAMBP levels are upregulated in human NSCLC tissues

**Fig. 1.**
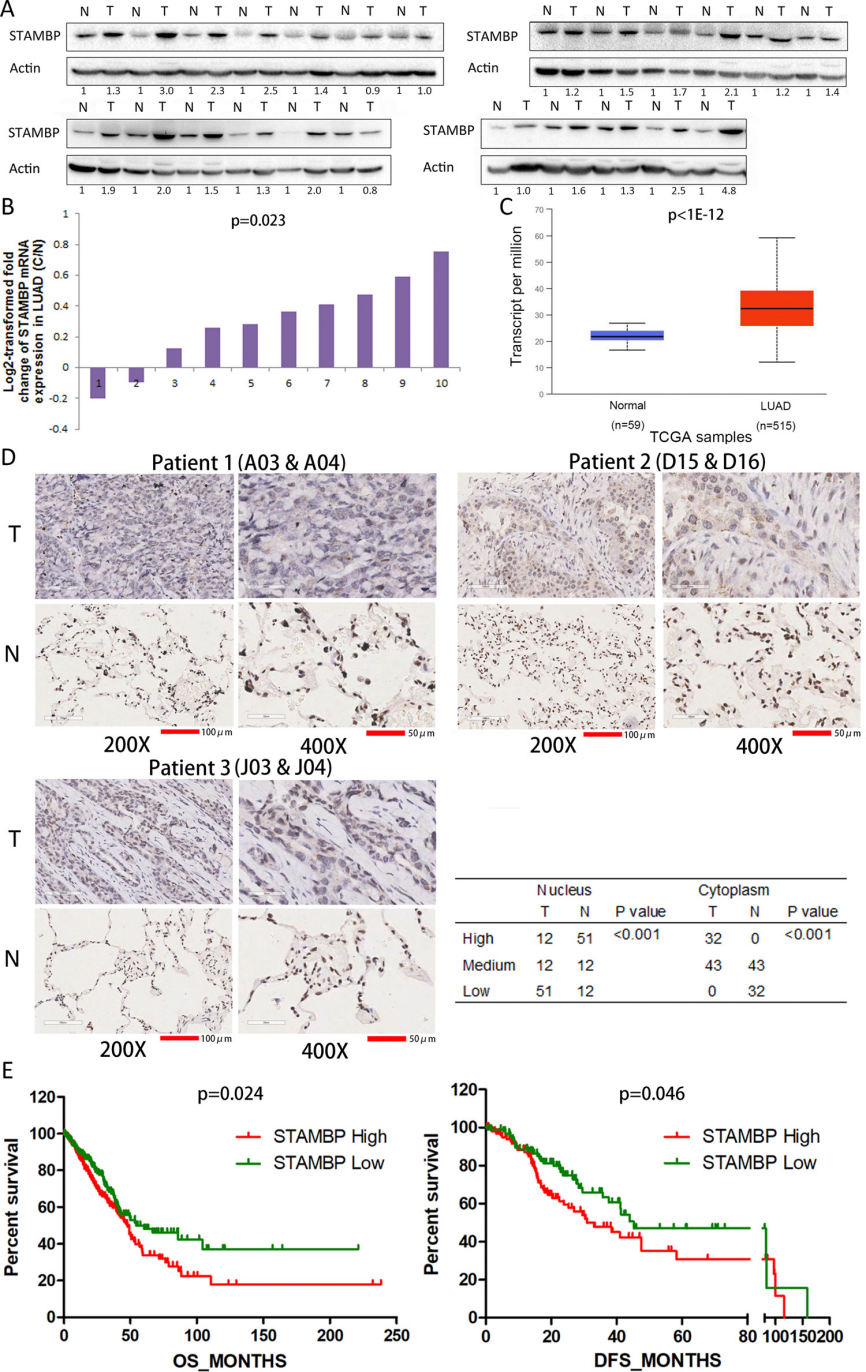
STAMBP expression is upregulated in NSCLC tissues. (A) The STAMBP protein expression in paired tumor tissues (T) and adjacent noncancerous tissues (N) from NSCLC patients was examined by Western blotting. β-Actin was used as the internal control. (B) The relative mRNA expression of STAMBP was analyzed in 10 paired tumor and noncancerous tissues from LUAD patients from the GEO database. The ratio of STAMBP expression in the tumor tissues to the controls for each patient is shown as a log2-transformed fold change. (C) Box (25−75th percentiles) and whisker (minimum-maximum) plots of STAMBP expression in the normal lung and tumor tissues from LUAD patients; the horizontal line inside the box indicates the median (the 50th percentile). *P* values were calculated by the Kruskal-Wallis test. (D) Typical IHC staining of STAMBP in tumor and noncancerous tissues. IHC staining was performed in 75 pairs of tissues, and representative images from 3 pairs (A03 and A04; D15 and D16; J03 and J04) are shown. Scale bar represents 100 µm (200 ×) and 50 µm (400 ×). The IHC scores were assessed, and the chi-squared test was used to compare the differences. (E) Kaplan-Meier survival analyses were conducted to evaluate the prognostic significance of STAMBP for OS and DFS in LUAD patients. NSCLC, non-small cell lung cancer; LUAD, lung adenocarcinoma; OS, overall survival; DFS, disease-free survival.

STAMBP plays critical roles in various physiological and pathological processes [22,26], but its involvement in tumor progression remains unclear. To explore STAMBP expression in NSCLC tissues, we detected the STAMBP protein in paired tumor and adjacent noncancerous tissues from 24 NSCLC patients. The results showed that STAMBP expression was upregulated in 20 patients, unchanged in 2 patients and downregulated in 2 patients (Fig. 1A). The STAMBP level was significantly increased in the NSCLC tissues compared with the noncancerous tissues (*P* < 0.01 by Fisher's exact test). We further assessed the STAMBP mRNA levels using publicly available GEO and TCGA databases. The STAMBP mRNA level was markedly elevated in 8 tissues and decreased in 2 tissues out of 10 LUAD tumor tissues compared with the paired noncancerous tissues from the GEO database (Fig. 1B). We also evaluated the STAMBP mRNA level in normal lung and tumor tissues of NSCLC patients using TCGA database. The STAMBP levels were significantly elevated in the tumor tissues from NSCLC patients (Fig. 1C and Supplementary figure 1). Next, we examined STAMBP expression and its cellular localization by IHC analysis using a tissue microarray of a large cohort of 75 LUAD patients. STAMBP expression was detected in both the tumor and noncancerous tissues (Fig. 1D and Supplementary figure 2). STAMBP staining was markedly increased in the cytoplasm of the tumor cells compared with that of the lung epithelial cells from noncancerous tissues. Stronger STAMBP staining was observed in the cytoplasm of the tumor cells from 32 out of 75 patients (42.7%). Surprisingly, the cytoplasm of the lung epithelial cells did not exhibit stronger STAMBP staining (Fig. 1D). STAMBP appears as discrete spots distributed in a speckled pattern in the cytoplasm of tumor cells. In contrast, STAMBP expression in the nuclei of tumor cells was downregulated in 51 patients (68%), unchanged in 12 patients (16.0%) and upregulated in 12 patients (16.0%) out of 75 patients compared with the corresponding noncancerous epithelial cells (Fig. 1D).

### STAMBP expression is associated with clinical features of LUAD patients

We evaluated the relationship between the expression of STAMBP and the clinical characteristics of NSCLC patients using the TCGA database. The results showed that STAMBP expression was associated with tumor size, lymph node invasion and neoplasm disease stage in the LUAD patients (Table 1). Although STAMBP expression was associated with tobacco smoking, there was no significant correlation between the STAMBP level and clinical features in LUSC patients (Supplementary table 3). Kaplan-Meier survival analysis was used to determine the prognostic impact of STAMBP on NSCLC patients. The results showed that LUAD patients with lower STAMBP expression had significantly longer OS and DFS than patients with higher STAMBP expression (Fig. 1E). The median OS and DFS were 47.77 and 30.98 mo for patients with higher STAMBP expression and 54.30 and 45.27 mo for patients with lower expression, respectively. In contrast, a high STAMBP level indicates a better OS but has no correlation with DFS in LUSC patients (Supplementary figure 3), suggesting that STAMBP could play different roles in the progression of the diseases.

**Table 1:** The expression of STAMBP was correlated with the demographic and clinicopathological characteristics of LUAD patients.

	STAMBP High	STAMBP Low	*P* value
Age			
≥60	173	172	0.860
<60	65	67	
Sex			
Male	116	115	0.924
Female	132	133	
Tobacco smoking history			
Stage3−5	146	150	0.708
Stage1−2	95	91	
Other malignancy history			
Negative	208	204	0.632
Positive	40	44	
Laterality			
Left	88	105	0.123
Right	152	136	
Location of lung parenchyma			
Peripheral lung	61	61	1.000
Central lung	31	31	
Residual tumor			
Negative (R0)	162	169	0.122
Positive (R1/R2)	11	5	
Tumor size			
T1	69	94	0.017^a^
T2-T4	178	153	
Lymph node invasion			
Negative	148	171	0.029^a^
Positive	96	73	
Distant metastasis			
Negative	161	169	0.153
Positive	16	9	
Neoplasm Disease Stage			
I/II	180	205	0.005^a^
III/IV	66	40	

The clinical information from NSCLC patients was retrieved from cBioportal for Cancer Genomics (TCGA) databases for correlation analysis. NSCLC: non-small-cell lung carcinoma; LUAD: lung adenocarcinoma.

a *P* < 0.05 was considered significant. The cutoff value for STAMBP expression was arbitrarily set at 4 and 7 patients were excluded from this analysis in LUAD patients. There existed some missing demographic and clinical data from each category in TCGA database. We analyzed all complete data acquired for each parameter from database.

### STAMBP promotes EGFR stabilization

**Fig. 2.**
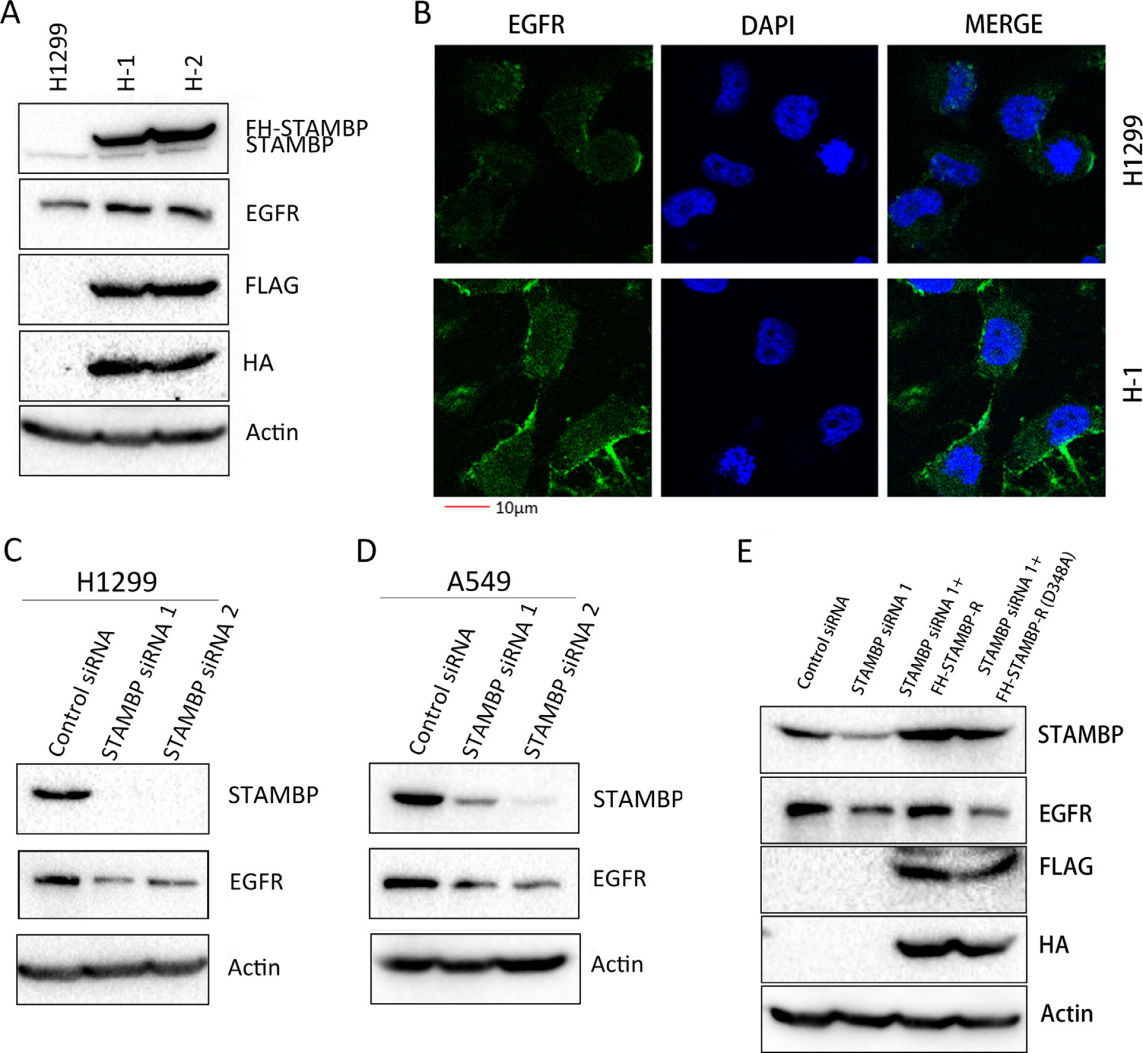

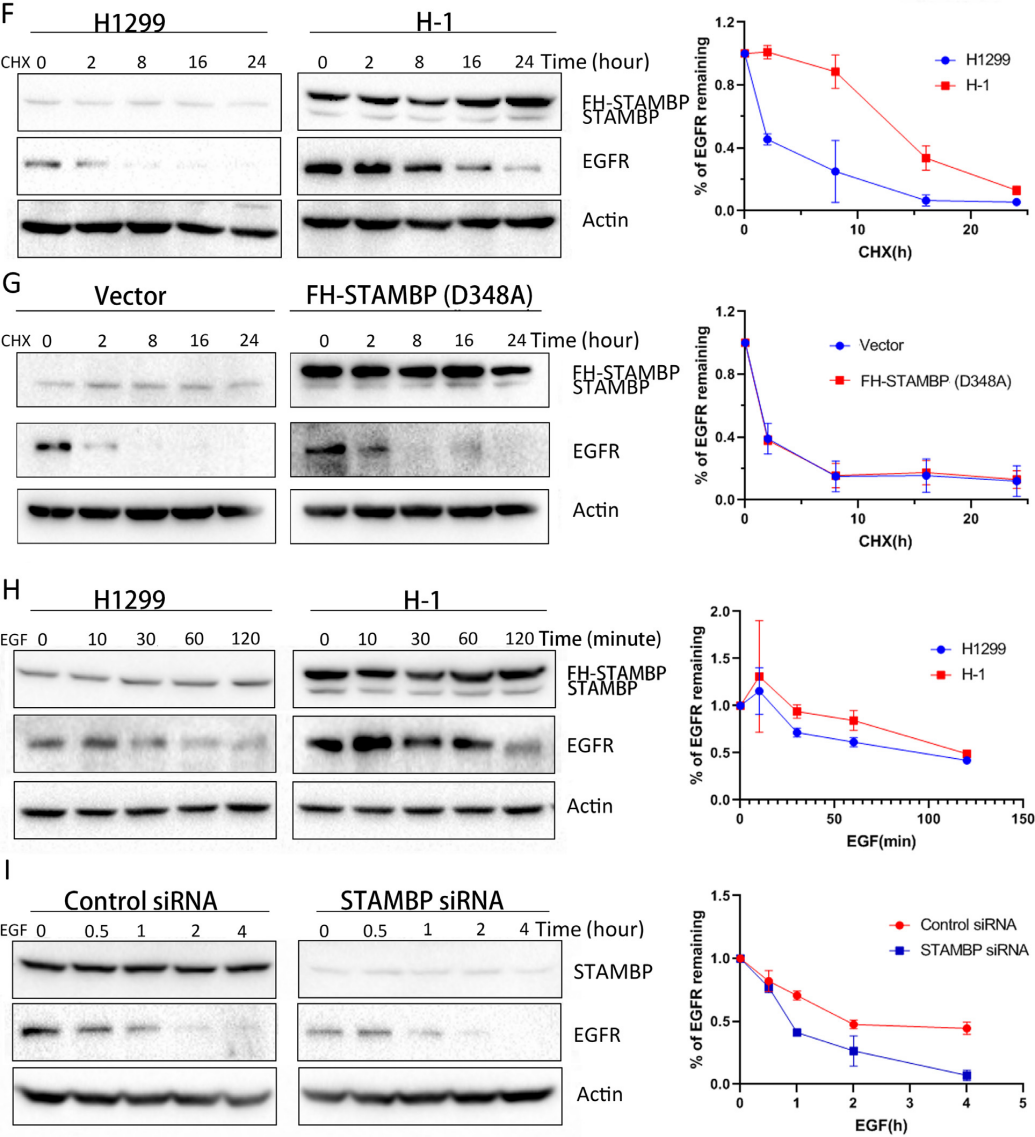
STAMBP promotes EGFR stabilization. (A) Western blot analysis of the cell extracts from the parental H1299 cells and 2 cell lines overexpressing STAMBP (H-1 and H-2) with the indicated antibodies. (B) H1299 and H1 cells were stained with EGFR primary antibodies followed by Alexa Fluor 488 (green) donkey anti-rabbit secondary antibodies. DAPI was used for the nuclear staining. The representative images of EGFR and merged staining are shown. (C and D) Western blot analysis of the cell extracts from the H1299 and A549 cells transfected with control siRNA, STAMBP-siRNA 1 and STAMBP-siRNA 2 with the indicated antibodies. (E) Western blot analysis of the cell extracts from the H1299 transfected with control siRNA, STAMBP-siRNA 1 without or with FH-STAMBP-R or FH-STAMBP-R (D348A) constructs with the indicated antibodies. (F) The parental H1299 and H-1 cells were treated with 10 μg/ml CHX for indicated times. (G) H1299 cells transfected with the empty vector or FH-STMABP (D348A) constructs were treated with 10 μg/ml CHX for indicated times. (H) The parental H1299 and H-1 cells were stimulated with 100 ng/ml EGF for the indicated times. (I) H1299 cells transfected with control siRNA or STAMBP-siRNA 1 were treated with 100 ng/ml EGF after 48 h and collected at the indicated times. Western blot analysis of the cell extracts was performed with the indicated antibodies (F-I). The experiments were repeated 3 times and quantification of the EGFR level relative to the β-actin level is shown in the right panel (F-I). DAPI, phenylindole dihydrochloride.

Although a previous report indicated that STAMBP promotes the stabilization of EGFR by deubiquitinating and recycling EGFR to the cell membrane [10], subsequent studies revealed that STAMBP contributes to EGFR degradation by deubiquitinating the cargo in MVB/late endosomes [19,30,31]. To investigate the effects of STAMBP on EGFR stability, we established stable H1299 cell lines overexpressing STAMBP, named H-1 and H-2. We observed that the EGFR levels were markedly increased in the H-1 and H-2 cells upon STAMBP overexpression (Fig. 2A). Furthermore, EGFR expression was mainly observed in cell membrane and stronger in H1 cells compared with parental H1299 cells by immunofluorescence staining (Fig. 2B). We used 2 specific siRNAs to knock down STAMBP expression in the H1299 and A549 cells. The EGFR levels were decreased following STAMBP knockdown in both cell lines (Fig. 2C and D). To convince the specificity of STAMBP siRNA targeting, we performed a rescue experiment by overexpressing siRNA resistant STAMBP constructs (FH-STAMBP-R), in which we mutated several nucleotides targeted by STAMBP siRNA but did not change amino acid sequences of the protein (Supplementary figure 4). We also overexpressed siRNA resistant STAMBP constructs expressing catalytically inactive STAMBP by replacing aspartic acid with alanine at codon 348 (GAC to GCC) of the protein, FH-STAMBP-R (D348A), to rescue the EGFR degradation following STAMBP knockdown. The results demonstrated that overexpression of FH-STAMBP-R but not FH-STAMBP-R (D348A) protected EGFR from degradation following STAMBP knockdown (Fig. 2E). The EGFR mRNA levels remained unchanged after STAMBP was overexpressed or knocked down from these cells (Supplementary figure 5), suggesting that the regulation occurs in a posttranscriptional manner. To determine whether STAMBP overexpression affects protein stability, we examined the half-life of EGFR after the cells were treated with cycloheximide (CHX) to block protein synthesis. The results demonstrated that the STAMBP levels remained constant even after the cells were treated with CHX for 24 h. Importantly, the EGFR protein stability was significantly increased in the H-1 cells (Fig. 2F). We transiently transfected the control and FH-STAMBP (D348A) constructs into H1299 cells to assess EGFR protein stability upon CHX treatment. The results showed that EGFR stability did not change when FH-STAMBP (D348A) was overexpressed in these cells (Fig. 2G), suggesting catalytic activity of STAMBP is crucial in maintaining EGFR protein stability. Upon EGF stimulation, EGFR is ubiquitinated and degraded by an endosome-lysosome pathway [26]. We also examined the half-life of EGFR after the cells were treated with EGF. The results demonstrated that increased STAMBP expression promoted EGFR stabilization, whereas decreased STAMBP expression accelerated EGFR degradation (Fig. 2H and I).

### STAMBP deubiquitinates EGFR by localizing in early endosomes

**Fig. 3.**
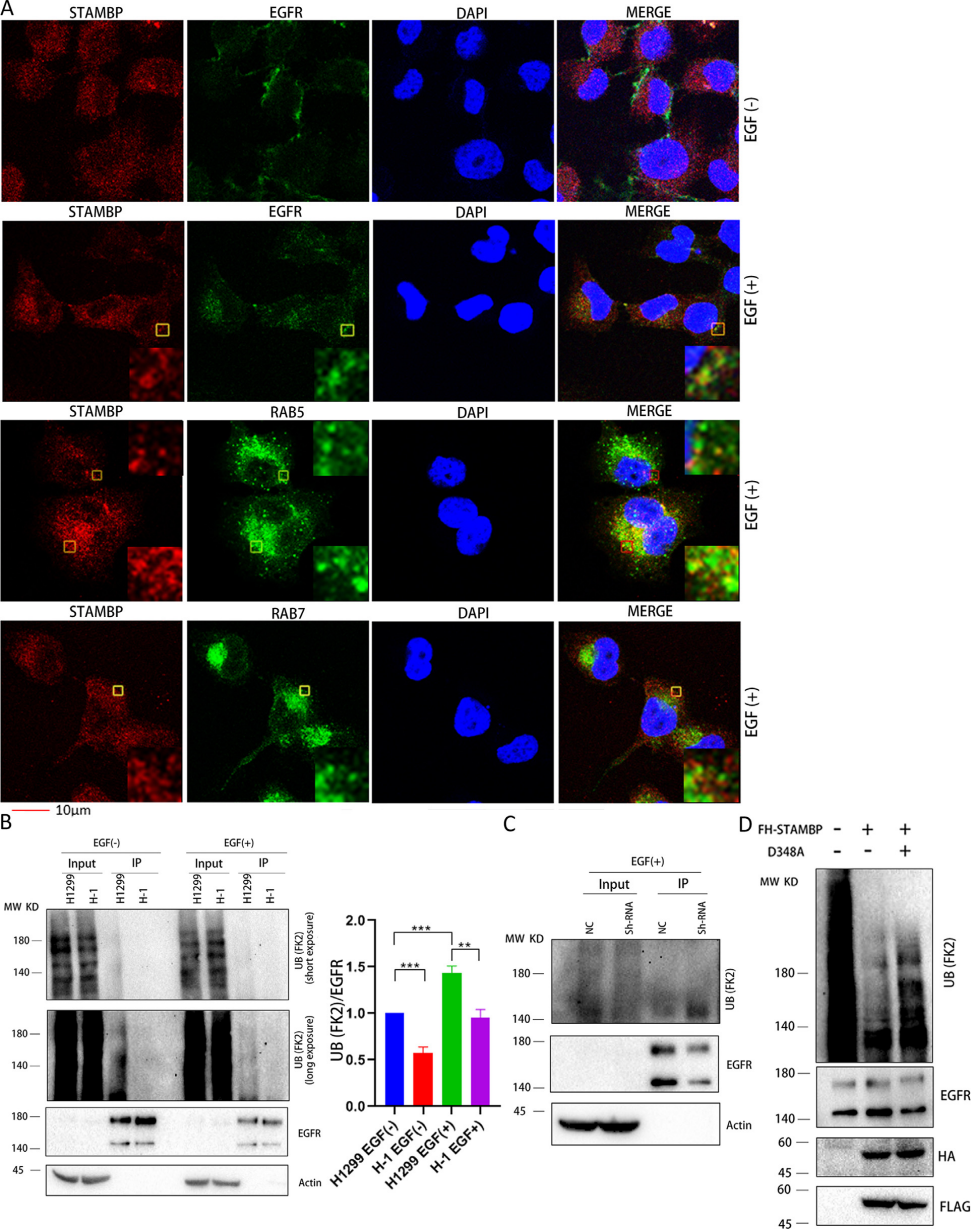
STAMBP deubiquitinates EGFR by localizing in early endosomes. (A) H1 cells were left untreated or treated with 100 ng/ml EGF for 20 min. The cells were stained with indicated primary antibodies followed by Alexa Fluor 488 (green) donkey anti-rabbit and Alexa Fluor 555 (red) donkey anti-mouse secondary antibodies. DAPI was used for the nuclear staining. The representative images of STAMBP, EGFR, RAB5, RAB7, DAPI and merged staining are shown. All panels show a single confocal section. Insets show 6-fold magnification of the boxed area. The scaled bar represents 10 µm. (B) H1299 and H1 cells were left untreated or treated with 100 ng/ml EGF for 15 min. (C) A549 cells stably expressing control and STAMBP shRNA were treated with 100 ng/ml EGF for 15 min. The cell lysates (B and C) were immunoprecipitated with anti-EGFR antibody and protein G agarose followed by western blot with anti-ubiquitin antibody (upper panel). The crude cell extracts were also detected with anti-EGFR and anti-β-Actin antibodies (lower panel). Relative ubiquitinated EGFR signal (ubiquitinated EGFR/total EGFR) was quantified from 3 repeated experiments and shown in right panel (B). (D) Purified EGFR complex was incubated with FH-STAMBP and FH-STAMBP (D348A) in a deubiquitination buffer. The reaction mixture was resolved by SDS-PAGE for western blot with anti-ubiquitin antibody (upper panel) as well as anti-EGFR, anti-HA and anti-FLAG antibodies (lower panel). DAPI, phenylindole dihydrochloride.

To provide mechanical insight into STAMBP-mediated EGFR protein stabilization in LUAD cells, we assessed whether STAMBP can bind directly to EGFR. The results demonstrated that STAMBP can directly bind to STAM but not EGFR (Supplementary figure 6), suggesting that the regulation occurs in an indirect manner. Although we cannot detect EGFR in STAMBP complex in H1299 cells, we found that STAMBP may co-localize with EGFR in speckled structures in perinuclear cytoplasm upon EGF treatment by immunofluorescence staining (Fig. 3A). We also investigated STAMBP co-localization with early or late endosome markers in these cells. The results demonstrated that STAMBP may co-localize with early endosome marker RAB5 but not, if any, with late endosome marker RAB7 upon EGF treatment (Fig. 3A). Next, we examined EGFR polyubiquitination in STAMBP overexpression and knockdown cells with or without EGF treatment. The results demonstrated that overexpression of FH-STAMBP decreased EGFR polyubiquitination (Fig. 3B) whereas STAMBP knockdown promoted EGFR polyubiquitination upon EGF treatment (Fig. 3C). We also performed *in vitro* deubiquitination assay by incubating EGFR complex with purified FH-STAMBP or FH-STAMBP (D348A) in a deubiquitination reaction. FH-STAMBP but not FH-STAMBP (D348A) may cleave polyubiquitination chain from EGFR (Fig. 3D). These results support STAMBP role in recycling EGFR to membrane by localizing in early endosomes and deubiquitinating EGFR.

### STAMBP overexpression promotes cell motility and invasion by activating MAPK signaling

**Fig. 4.**
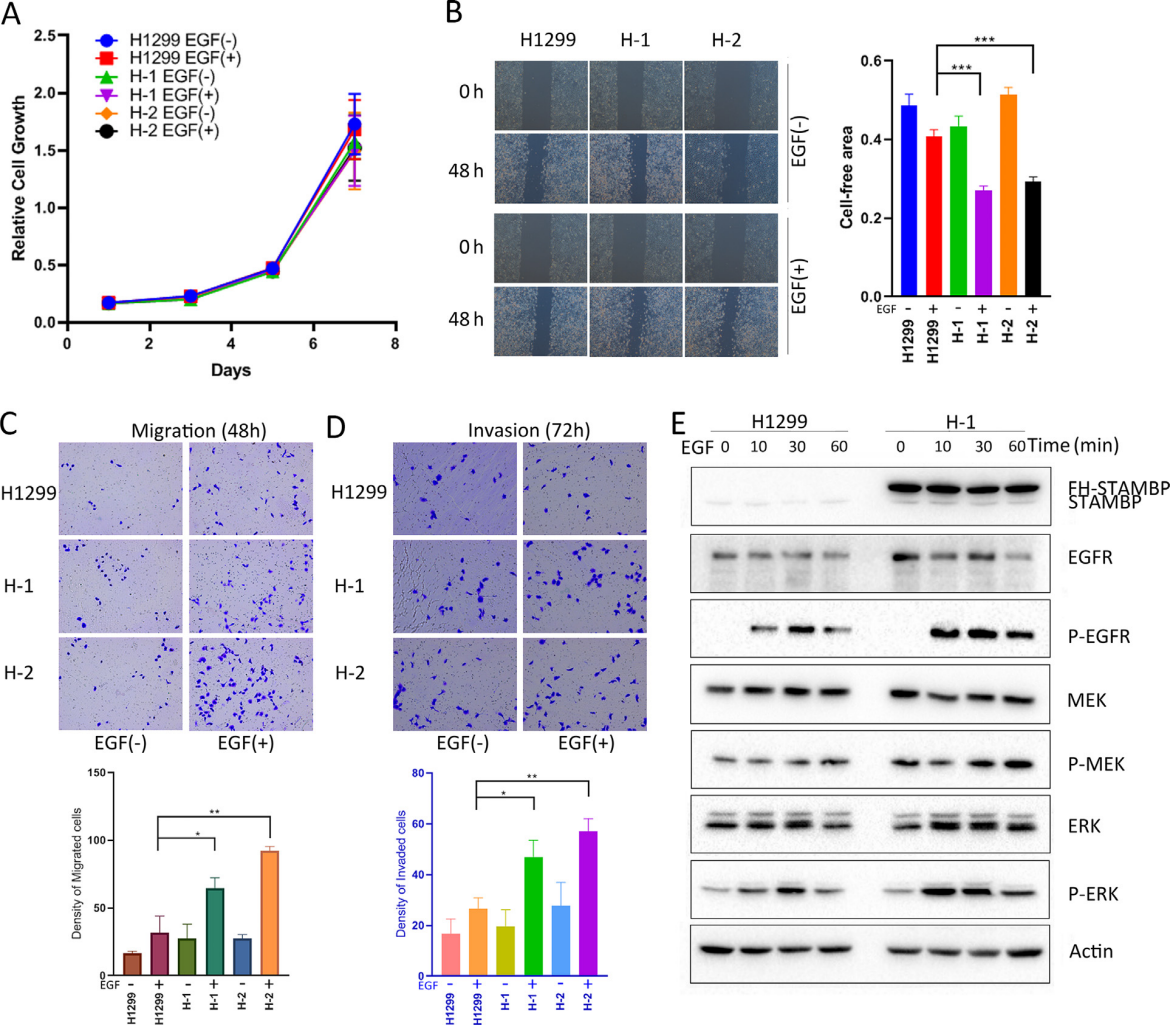
STAMBP overexpression promotes cell motility and invasion by activating MAPK signaling. H1299, H-1 and H-2 cells were stimulated with or without 100 ng/ml EGF for the indicated times. (A) Cell growth was determined at the indicated times. (B) A wound healing assay was performed, and wound healing was monitored at the indicated time points. (C and D) The migrating and invading cells were observed and counted at indicated times. (E) Western blot analysis of the cell extracts with indicated antibodies. The experiments were repeated twice, and a representative result is shown (B-D).

EGFR is frequently overexpressed in NSCLC and triggers the MAPK signaling cascade to induce tumor growth and metastasis [9]. Due to EGFR stabilization by STAMBP, we investigated the role of STAMBP in the progression of the disease. We used H1299 and A549 cells to assess the effects of STAMBP on cell growth, mobility and invasion. The results showed that STAMBP overexpression did not affect the growth of cells treated with or without EGF (Fig. 4A). Next, we performed a wound healing assay to assess the effects of STAMBP on cell mobility. The results demonstrated that the H1 and H2 cells showed stronger mobility than the parental H1299 cells after EGF treatment (Fig. 4B). We also examined the effects of STAMBP on cell migration and invasion using the Transwell detection system. Consistently, the migration and invasion abilities of the H1 and H2 cells were significantly stronger than that of the H1299 cells after EGF treatment (Fig. 4C and D). EGFR, MEK and ERK phosphorylation was enhanced, whereas the MEK and ERK levels remained unchanged in the H1299 cells after EGF treatment. Importantly, the EGFR levels were markedly increased, and the phosphorylation cascade was more significantly activated in the H1 cells than in the parental H1299 cells (Fig. 4E).

### STAMBP knockdown inhibits cell motility and invasion by attenuating MAPK signaling

**Fig. 5.**
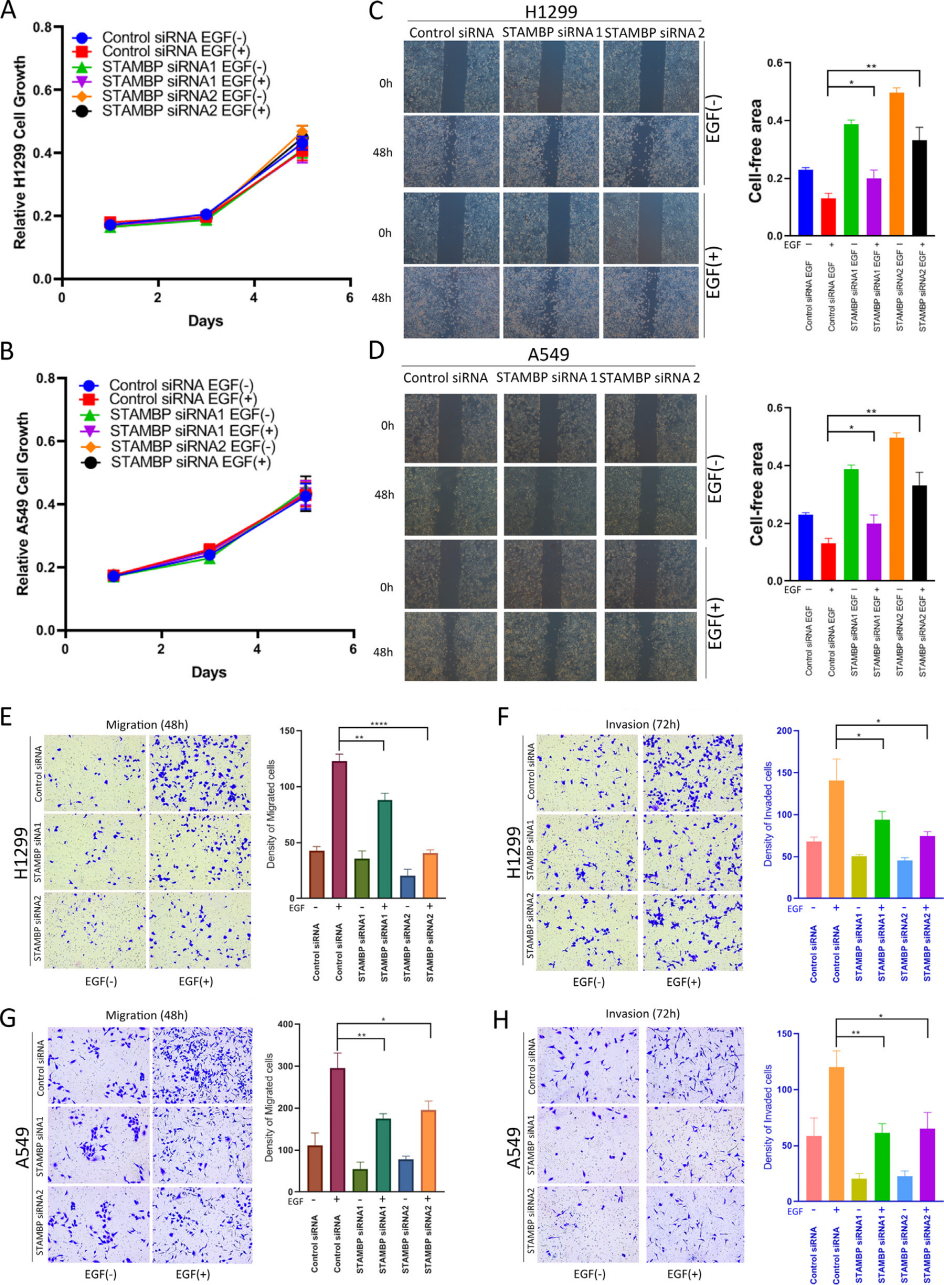

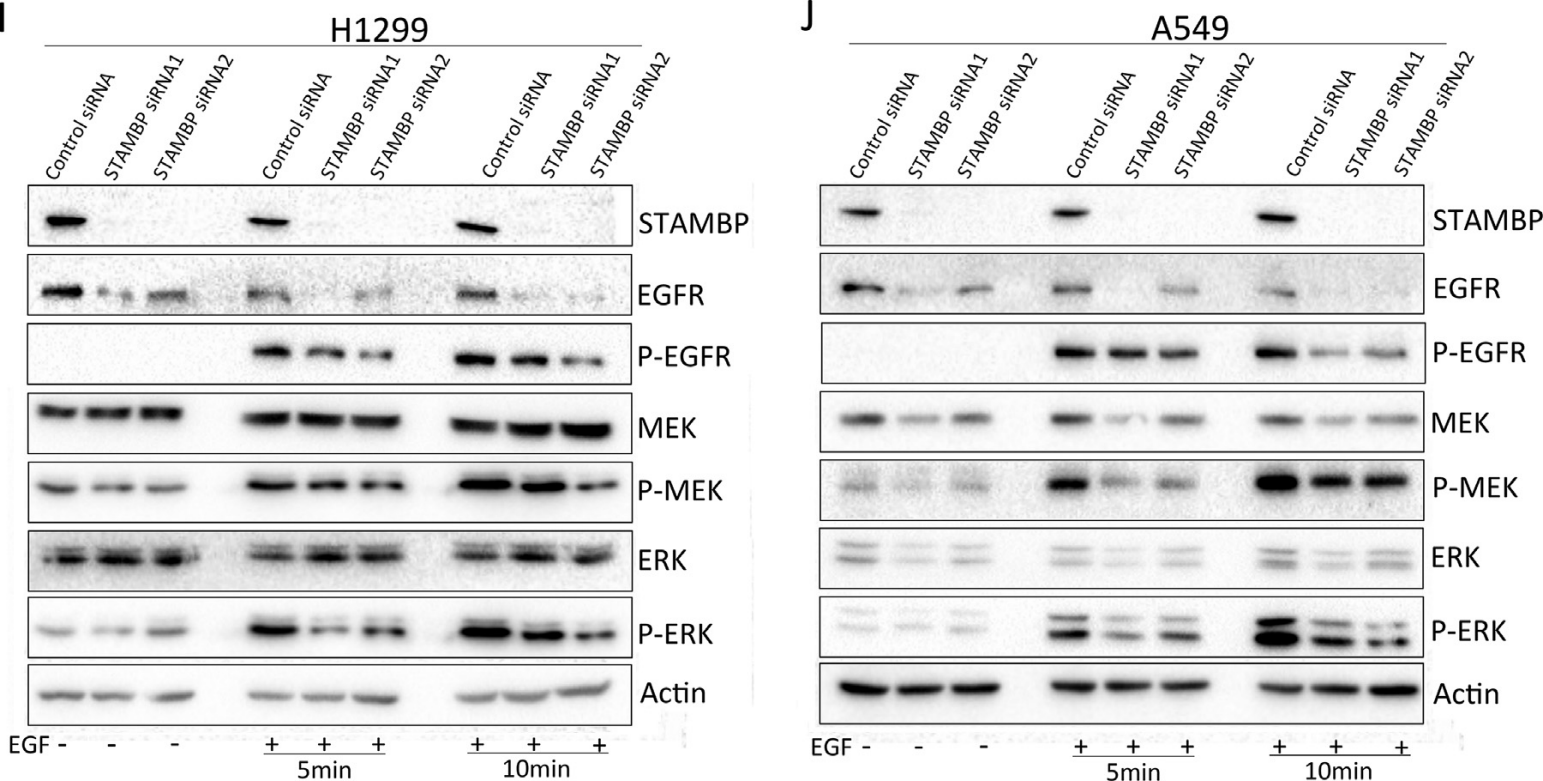
Knockdown of STAMBP inhibits cell motility and invasion by attenuating MAPK signaling. H1299 and A549 cells transfected with control siRNA, STAMBP-siRNA 1 and STAMBP-siRNA 2 were treated with or without EGF (100 ng/ml). (A and B) Cell growth was measured at the indicated time points. (C and D) A wound healing assay was performed, and wound healing was monitored at the indicated time points. (E and F) Migrating and invading H1299 cells were observed and counted after 48 and 72 h, respectively. (G and H) The migrating and invading A549 cells were observed and counted after 72 and 48 h, respectively. (I and J) Western blot analysis of cell extracts with indicated antibodies. The experiments were repeated twice, and a representative result is shown (C-H).

We used 2 siRNA fragments to reduce STAMBP expression in H1299 and A549 cells and then examined the ability of the cells to proliferate, migrate and invade. The results showed that STAMBP knockdown did not affect the proliferation of H1299 and A549 cells treated with or without EGF (Fig. 5A and B). Wound healing was markedly delayed in the STAMBP knockdown cells after EGF stimulation but not in the untreated cells (Fig. 5C and D). Consistently, migration and invasion were also significantly attenuated in the STAMBP knockdown cells after EGF stimulation (Fig. 5E and F). Upon EGF stimulation, EGFR, MEK and ERK were phosphorylated and activated in the H1299 and A549 cells. The phosphorylation of these tyrosine kinases was markedly reduced when STAMBP expression was knocked down in these cells (Fig. 5G and H).

### The effects of STAMBP are blocked by small molecule inhibitors of the EGFR/MAPK signaling pathway

**Fig. 6.**
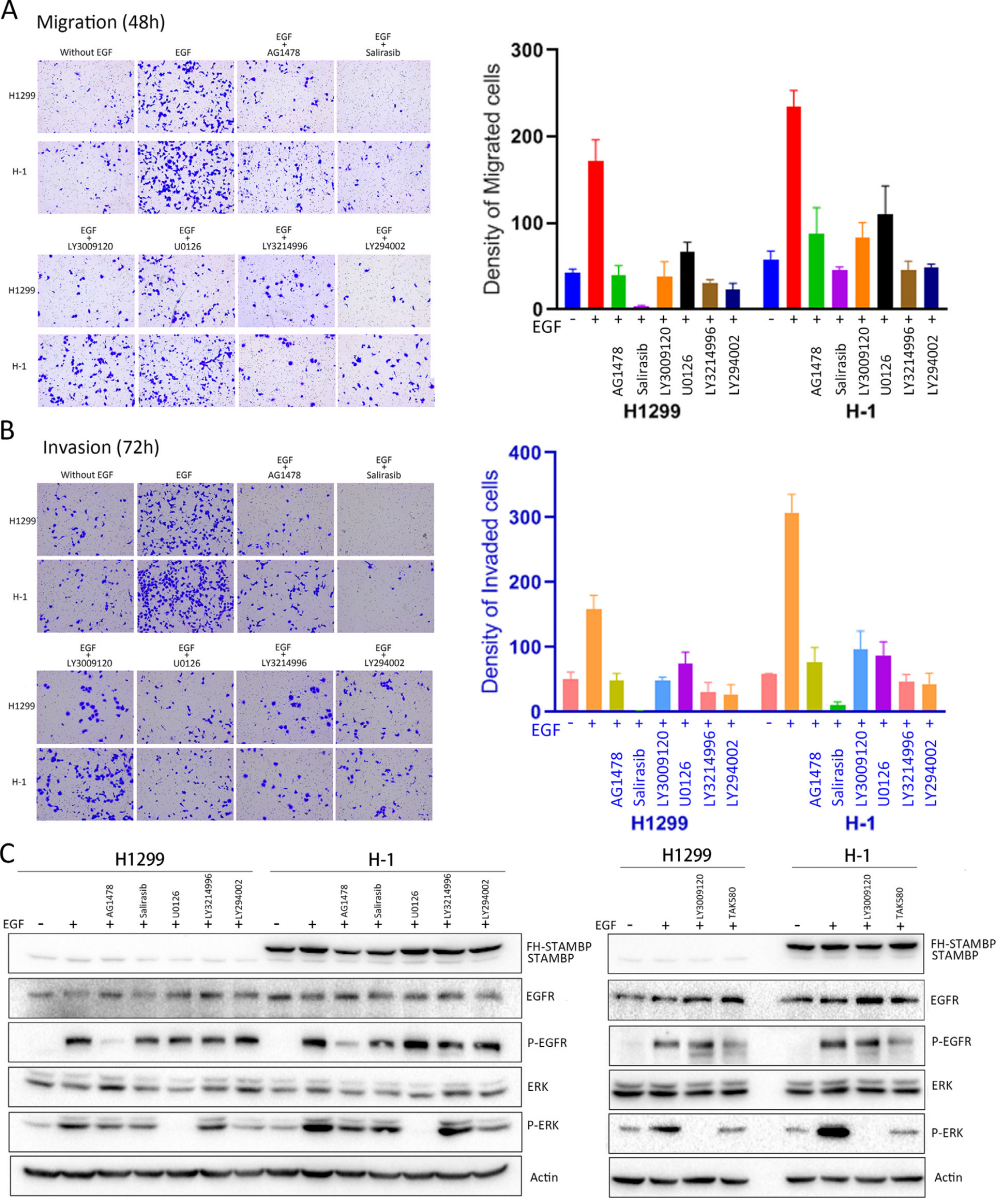
The effects of STAMBP are blocked by small molecule inhibitors of EGFR/MAPK signaling. (A-B) H1299 and H-1 cells were treated with or without 100 ng/ml EGF for 48 or 72 h. The cells were treated with or without the EGFR inhibitor AG1478 (4 μM), the RAS inhibitor salirasib (140 μM), the RAF inhibitors TAK580 (10 μM) and LY3009120 (10 μM), the MEK inhibitor U0126 (10 μM), the ERK inhibitor LY3214996 (10 μM), and the PI3K inhibitor LY294002 (5 μM). The migratory and invading cells on the underside of the filter were fixed, stained and counted. The experiments were repeated twice, and a representative result was shown. (C) H1299 and H1 cells were treated as indicated above for 24 h. Western blot analysis of cell extracts was performed with indicated antibodies.

We performed cell migration and invasion assays in the presence of the EGFR inhibitor AG1478, the Ras inhibitor salirasib, the Raf inhibitors TAK580 and LY3009120, the MEK inhibitor U0126, the ERK inhibitor LY3214996 and the PI3K/Akt pathway inhibitor LY294002. Inclusion of all the inhibitors prevented the migration and invasion of the parental H1299 cells and H1 cells (Fig. 6A and B). EGFR phosphorylation was markedly reduced after AG1478 treatment but remained unchanged when the cells were treated with EGF in the presence of the other inhibitors (Fig. 6C). The ERK phosphorylation induced by EGF treatment was blocked by all these inhibitors in the H1299 and H1 cells. These results suggested that the cell migration and invasion induced by STAMBP overexpression were mediated by the activation of EGFR and the RAS/RAF/MEK/ERK signaling pathway. BC-1471, a small molecular inhibitor against STAMBP catalytic activity, may decrease NALP7 protein levels by blocking STAMBP deubiquitinase activity [26]. We assessed BC-1471 effects on STAMBP deubiquitination activity against EGFR and on the stability of EGFR in LUAD cells. We cannot observe its effects on STAMBP-mediated deubiquitination of EGFR in vitro and the stability of EGFR in LUAD cells (Supplementary figure 7A-C).

### STAMBP knockdown suppresses tumor growth and metastasis in vivo

**Fig. 7.**
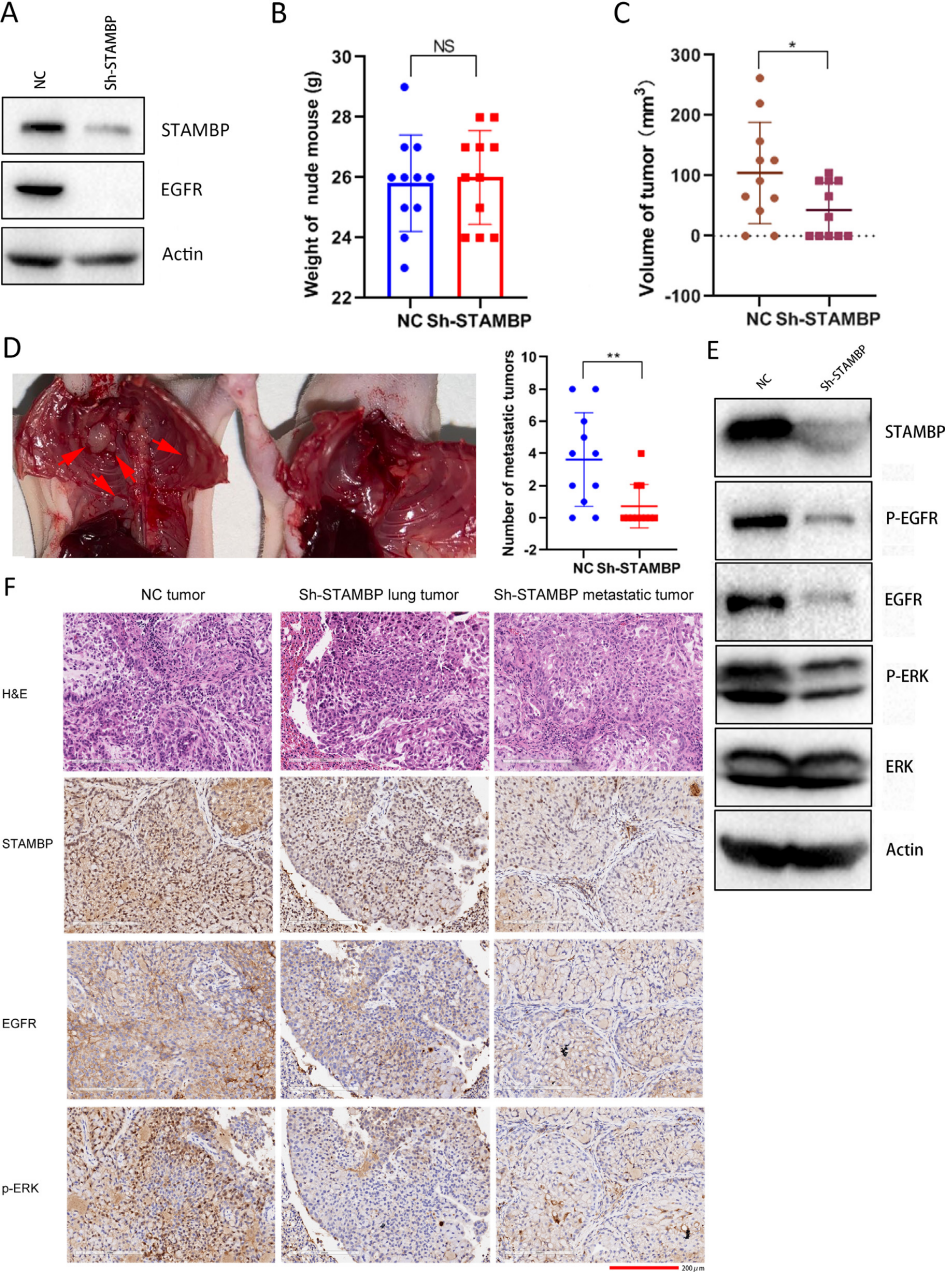
STAMBP knockdown suppresses tumor growth and metastasis in vivo. (A) Western blot analysis of cell extracts from A549 cells stably expressing control and STAMBP shRNA with indicated antibodies. (B) The weight of the nude mice with tumor inoculation on the day of sacrifice. (C) The tumor volumes were determined in the control and STAMBP knockdown groups on the day of sacrifice. (D) An image of the metastatic tumors in the chest wall is shown. The number of metastatic tumors was compared between the control and STAMBP knockdown groups. (E) Western blot analysis of cell extracts from lung tumors with indicated antibodies. (F) Representative IHC staining of STAMBP, EGFR and p-ERK in the lung tumors and metastatic tumors in the chest walls from the control and STAMBP knockdown tissues. Scale bar represents 200 µm.

To assess the effect of STAMBP on tumor growth and metastasis in vivo, we established A549 cells with stable STAMBP knockdown by lentivirus-mediated shRNA expression. STAMBP and EGFR expression was markedly decreased in these cells (Fig. 7A). Consistently, cell migration and invasion were largely compromised in STAMBP knockdown cells after EGF stimulation (Supplementary figure 8A and B). EGFR and ERK phosphorylation was inhibited after STAMBP was knocked down after EGF treatment (Supplementary figure 8C). The control and STAMBP knockdown cells were separately injected into the left lungs of nude mice. After 6 wk of feeding, the mice were euthanized and weighed, the lungs were removed, and chest wall metastases were observed. All the nude mice grew healthy, and there was no difference in the body weight between the 2 groups (Fig. 7B). The tumors in the lungs of 9 out of 11 nude mice from the control group were observed. All 9 mice showed different numbers of metastatic tumors on the chest wall. However, only 6 out of 11 nude mice from the STAMBP knockdown group developed a tumor on the left lung and 3 out of 6 mice formed metastatic tumors in the chest wall. The volume of the tumors in the lung was smaller and the number of metastatic tumors in the chest wall was lower in the STAMBP knockdown group (Fig. 7C and D; Supplementary figure 9A). Next, we evaluated the protein expression in the tumor tissues by Western blot. The results showed that STAMBP, EGFR, phosphorylated EGFR and ERK were markedly reduced, whereas the ERK levels remained unchanged in the STAMBP knockdown tumor tissues (Fig. 7E). Finally, STAMBP expression and MAPK signaling activation in the tumors were evaluated by IHC staining. The xenograft tumor tissues with new blood vessels were clearly shown by H&E staining (Fig. 7F). STAMBP staining was mainly observed in the nucleus, and weak staining was observed in the cytoplasm of mouse lung epithelial cells (Supplementary figure 9B). In contrast, strong STAMBP staining was detected in the cytoplasm, and weak staining was detected in the nucleus of the tumor cells. Importantly, the expression of STAMBP, EGFR in cell membrane and phosphorylated ERK in cell nucleus was markedly reduced in the STAMBP knockdown tumor tissues from the lung and metastatic tumors from the chest wall (Fig. 7F).

## Discussion

Accumulating evidence implicates DUBs in tumorigenesis and progression at multiple levels [38]. Our study indicates that OTUD5, a member of the ovarian tumor protease (OTU) subfamily of DUBs, cooperates with TRIM25 in tumor suppression via deubiquitination activity [37]. The proteasome DUBs PSMD14 and UCHL5 are implicated in LUAD progression through the regulation of cell cycle proteins [36,39]. Here, we evaluated the expression and role of STAMBP in NSCLC progression. STAMBP expression is elevated in the cytoplasm of tumor cells and reflects the severity of the disease. STAMBP promotes cell migration and invasion in vitro, and STAMBP knockdown suppresses tumor growth and metastasis in a xenograft mouse model. Metastasis is a leading cause of cancer-related death among NSCLC patients [40,41]. The phosphorylation and activation of EGFR and the MAPK signaling cascade are thought to be key pathways involved in NSCLC metastasis [4−6]. Mechanistically, STAMBP may deubiquitinate EGFR by localizing in early endosomes and increase EGFR membrane localization in LUAD cells. STAMBP regulates tumor metastasis by increasing EGFR stability to trigger MAPK signaling. Our findings broaden the current understanding of the mechanism underlying NSCLC metastasis.

LUAD is molecularly classified as EGFR-mutant, KRAS-mutant and many other categories of driver alterations (fusions and mutations). Tumors with EGFR and KRAS mutations are mutually exclusive and the frequency of KRAS and EGFR mutations is each 10% to 30% [42]. Although EGFR-TKI treatment has shown excellent effects on EGFR mutation patients, acquired resistance has dampened long term benefit of these drugs. Targeting the other key signaling proteins or combinational treatment provide alternative strategies for LUAD patients. The multiple regulatory pathways are involved in EGFR signaling pathways. Recent studies indicate co-dependence signaling by SHOC2 signaling in either KRAS mutant or EGFR mutant lung cancer cells [43,44]. SHOC2 inhibitor cooperated with MEK inhibitor or EGFR-TKI impairs cell proliferation and viability in KRAS- or EGFR mutant driven tumor cells. SHOC2 may be a promising target for combination therapy for LUAD [43,44]. In addition, the Hippo effector YAP promotes resistance to RAF- and MEK-targeted tumor therapies [45]. YAP signaling is critical for SLUG activation and treatment-induced tumor dormancy through YAP-mediated transcriptional reprogramming of the apoptotic pathway [46]. Blocking YAP signaling pathway contributes to eliminate tumor cells upon EGFR-TKI or MEK inhibitor treatment.

The exploration of the molecular events that regulate EGFR degradation is critical for the development of new treatment strategies for EGFR-positive NSCLC patients. Although tyrosine kinase inhibitors (TKIs) that target mutant EGFR in NSCLC have been successful in controlling tumor growth, they may also invariably induce acquired resistance due to EGFR mutations [[47], [48], [49]]. Moreover, wild-type EGFR is overexpressed in 10% to 90% of NSCLC tumor tissues, whereas its activation mutation is only found in 10% of NSCLC patients [9]. A total of 10% to 20% of NSCLCs with EGFR mutations are resistant, and most NSCLCs with wild-type EGFR cannot respond well to TKIs despite their elevated EGFR expression [47−49]. Thus, the manipulation of EGFR stability is an alternative therapy. EGFR degradation has been induced by various strategies, including delivering EGFR-specific siRNAs [50,51], proteolysis-targeting chimera (PROTAC) technology [52] and small molecular drugs [53,54]. Our findings that STAMBP knockdown promotes EGFR degradation and suppresses LUAD tumor metastasis raise the possibility that targeting STAMBP may be a promising strategy to control LUAD progression. EGFR can be both mono- and polyubiquitinated mainly through Lys63 chains [55,56]. Although the role of 2 types of ubiquitination in EGFR endocytosis and/or signaling has not been completely revealed, accumulating evidence indicates that EGFR Lys63-linked polyubiquitination chains are critical for EGF induced EGFR sorting and targeting to the lysosome for degradation [4,7,8,55−57]. We found that EGFR was polyubiquitinated and its polyubiquitination level was markedly increased upon EGF treatment in H1299 cells. EGFR was degraded followed by EGFR signaling activation in LUAD cells upon EGF treatment. These findings support that polyubiquitination is critical for EGF-induced EGFR degradation and regulation of EGFR signaling in NSCLC.

The regulation of EGFR degradation is complicated, and the effect of STAMBP on EGFR remains controversial. The role of ubiquitination or deubiquitination in the sorting of receptor tyrosine kinases could be different in the physiological cellular process from that in pathological status (normal cell and tumor cell), which might lead to the controversial interpretation of STAMBP on EGFR regulation. Several studies indicate that STAMBP functions as an ESCRT-III-associated enzyme in late endosomes to promote EGFR degradation [19,30,31]. Consistently, STAMBP contributes to the degradation of various membrane components, including CXCR4, PAR2 and Cx43, via its localization in the ESCRT complex and endosomes [16,27,29]. Other studies found that STAMBP on early endosomes contribute to EGFR stabilization by recycling EGFR to the cell membrane [8,10]. A recent report supports this model by demonstrating that STAMBP impedes NALP7 trafficking to lysosomes to increase NALP7 abundance via deubiquitinase activity [26]. We found that STAMBP is overexpressed in NSCLC and promotes EGFR stabilization in vitro and in xenograft tissues. STAMBP expression is elevated in the cytoplasm of tumor cells, which provides further support for its roles in regulating EGFR stability and promoting EGFR recycling to the membrane. Our findings describe a unique pathway of EGFR regulation and identify STAMBP as a potential therapeutic target to suppress LUAD tumor metastasis.

## Appendix Supplementary materials

Image, application 1

Image, application 2

Image, application 3

Image, application 4
