# Supplementary material for: STAMBP promotes lung adenocarcinoma metastasis by regulating the EGFR/MAPK signaling pathway
Source: Neoplasia. 2021 Jun 5;23(6):607–23. doi: 10.1016/j.neo.2021.05.011 (PMC8190130; doi:10.1016/j.neo.2021.05.011)
Supplement: Supplementary file 1 [file mmc1.docx]

**List of Supporting Information:**

Table S1. The demographical and clinical features of 24 NSCLC patients

Table S2. The demographical and clinical features of 75 LUAD patients

Table S3. STAMBP expression and the demographic and clinical characteristics in LUSC patients

Figure S1. STAMBP expression is upregulated in LUSC tumor tissues

Figure S2. STAMBP expression was increased in LUAD tumor tissues

Figure S3. High STAMBP expression indicates a better OS in LUSC patients

Figure S4. Schematic diagram of FH-STAMBP and its mutants

Figure S5. STAMBP cannot regulate EGFR transcription in H1299 cells

Figure S6. STAMBP can bind to STAM but not to EGFR in H1299 cells

Figure S7. The effects of BC-1471 on STAMBP-mediated deubiquitination and stability of EGFR

Figure S8. STAMBP stable knockdown inhibits the migration and invasion of A549 cells

Figure S9. STAMBP knockdown suppresses tumor growth and metastasis in vivo

**Supplementary figure 1**

**
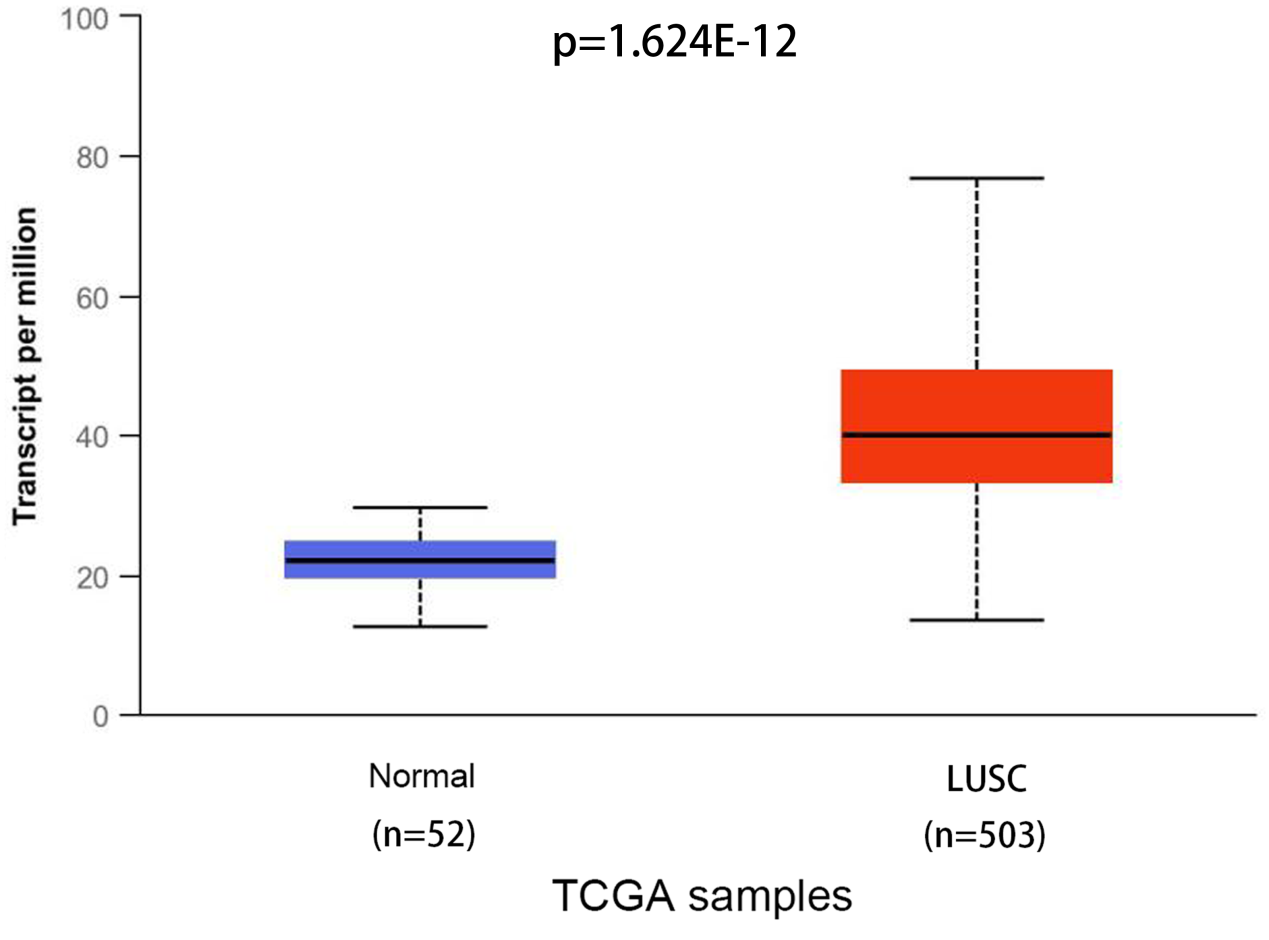
**

**Figure S1 STAMBP expression is upregulated in tumor tissues from LUSC patients.** Box (25-75th percentiles) and whisker (minimum-maximum) plots for STAMBP expression in normal lung tissues and tumor tissues from LUSC patients; the horizontal line inside the box indicates the median (the 50th percentile). P-values were calculated by the Kruskal-Wallis test. TCGA, The Cancer Genome Atlas; LUSC, lung squamous cell carcinoma.

**
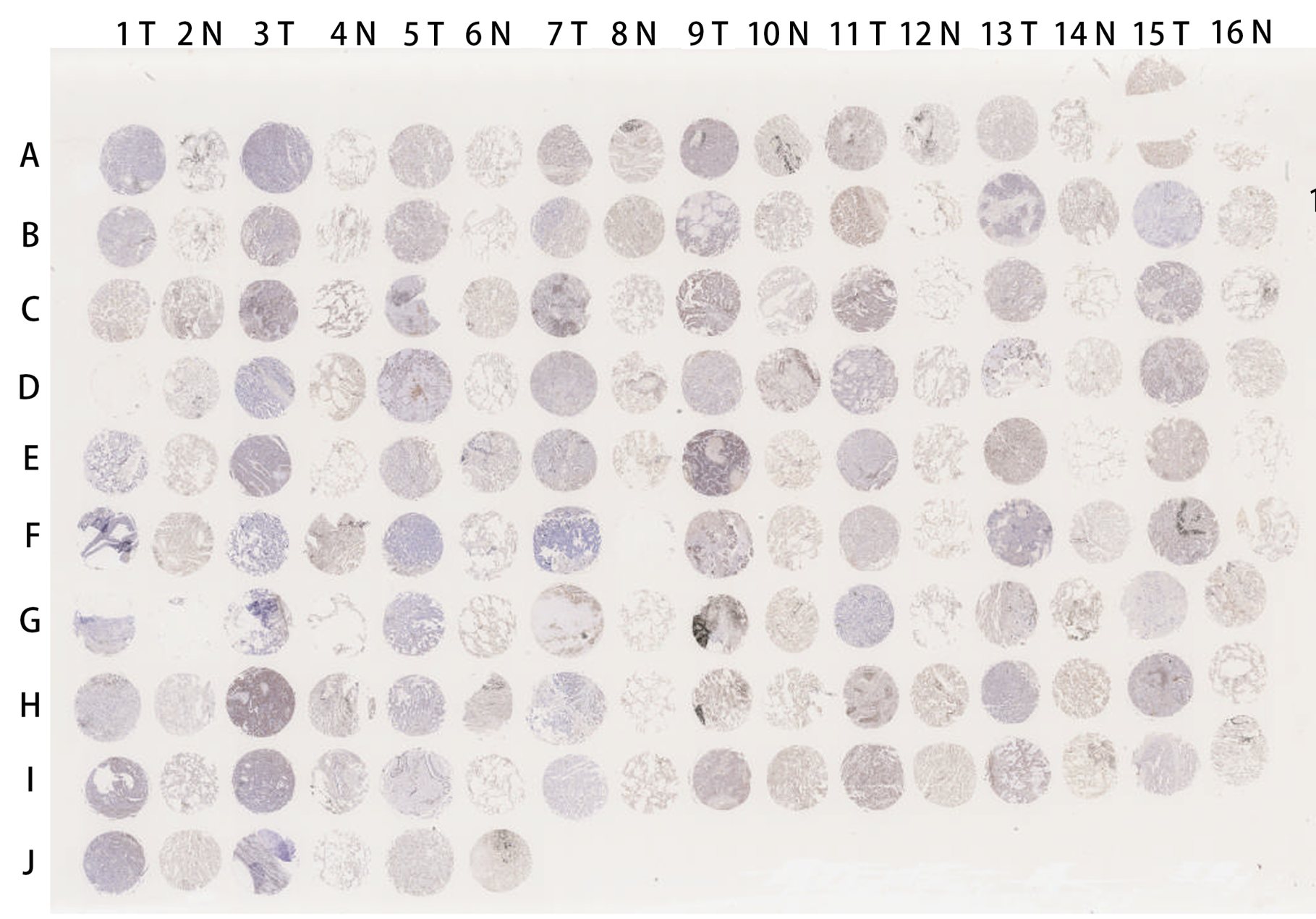
Supplementary figure 2**

**Figure S2 STAMBP expression was increased in cytoplasm of tumor tissues from LUAD patients.** STAMBP expression was determined by IHC staining in human LUAD tissue microarray including 75 pairs of lung cancer tissues (T) and adjacent non-cancerous tissues (N). IHC: immunohistochemistry; LUAD: lung adenocarcinoma.

**Supplementary figure 3**

**
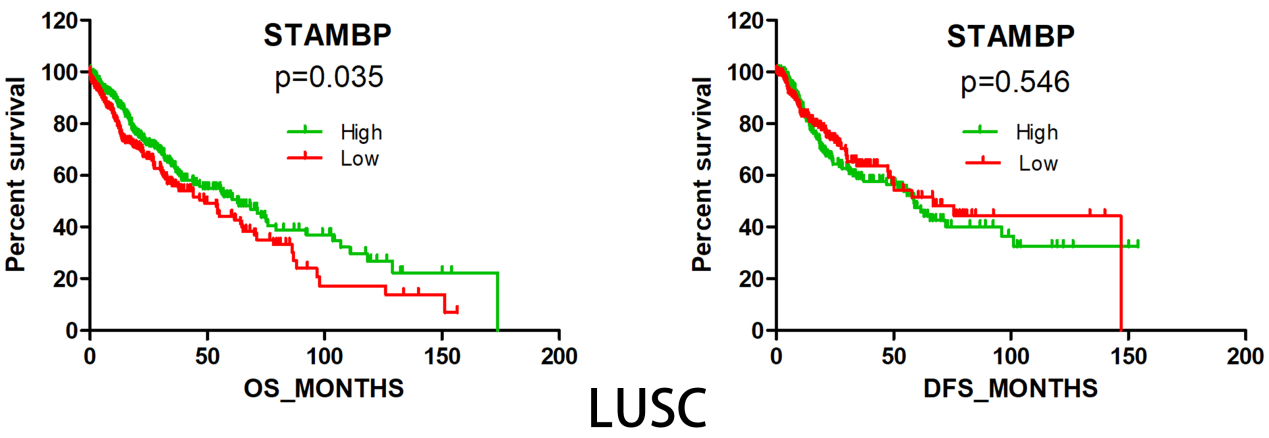
**

**Figure S3 High STAMBP expression indicates a better OS in LUSC patients.** We stratified 478 and 364 LUSC patients from TCGA database into higher and lower STAMBP expression levels for the overall survival (OS) and the disease-free survival (DFS) analyses. Survival curves were plotted using the Kaplan-Meier method and compared by the log-rank test. Statistical significance was set at p<0.05. TCGA, The Cancer Genome Atlas; LUSC, lung squamous cell carcinoma.

**Supplementary figure 4**


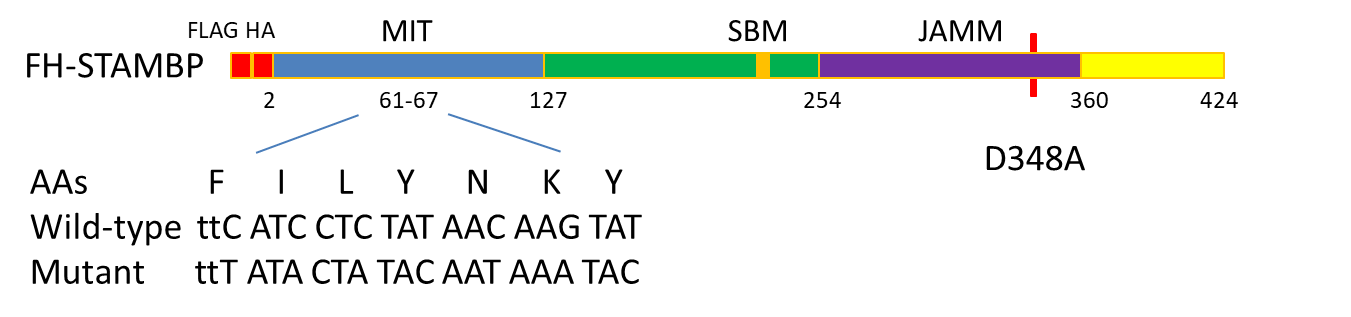


**Figure S4 Schematic diagram of FH-STAMBP and its mutants.** FH-STAMBP contains N-terminal FLAG and HA tags followed by a MIT domain, a SH3 binding motif (SBM) and a JAMM (JAB1/MPN/MOV34) motif 22. The STAMBP sequences targeted by siRNA 1 and mutated resistant sequences were shown as wild-type and mutant in capital letters, respectively. The corresponding amino acid residue sequences were also shown (AAs). FH-STAMBP-R constructs contain a siRNA resistant sequence. FH-STAMBP (D348A) constructs contain catalytically inactive D348A mutation. FH-STAMBP-R (D348A) constructs contain both a siRNA resistant sequence and catalytically inactive D348A mutation. All these mutants were generated from GenScript (Nanjing, Jiangsu, China) and confirmed by DNA sequencing.

**Supplementary figure 5**

**
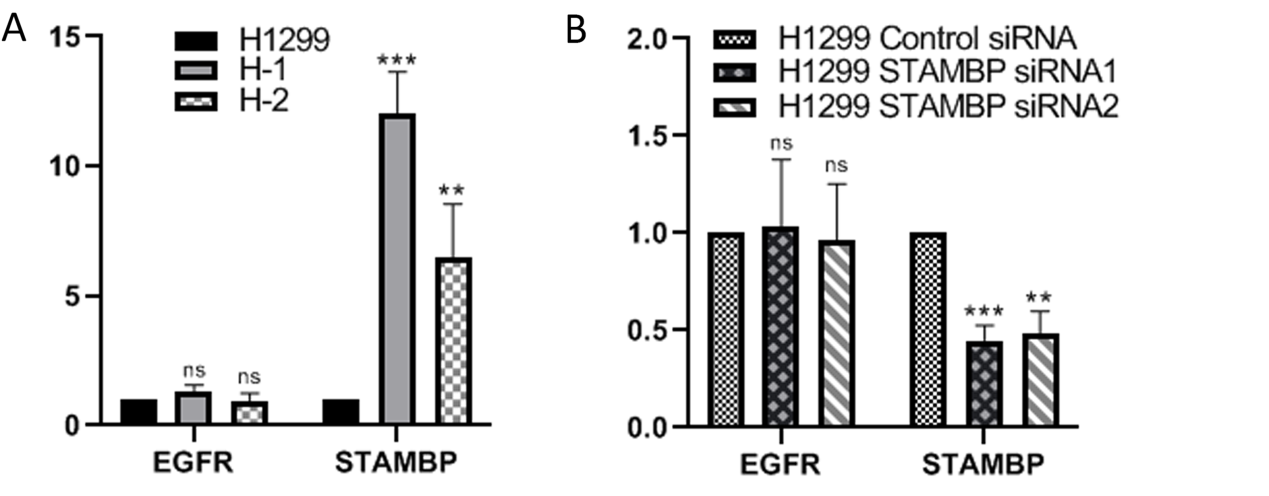
**

**Figure S5 STAMBP cannot regulate EGFR mRNA transcription in H1299 cells.** (A) The mRNA expression of STAMBP and EGFR in parental H1299 and H-1 and H-2 overexpressing STAMBP was determined by Real-time PCR. β-Actin was used as a control. (B) The mRNA expression of STAMBP and EGFR in H1299 transfected with control siRNA, STAMBP siRNA1 and STAMBP siRNA2 was determined by Real-time PCR. β-Actin was used as a control. The experiments were repeated three times and a representative picture was shown.

**Supplementary figure 6**

**
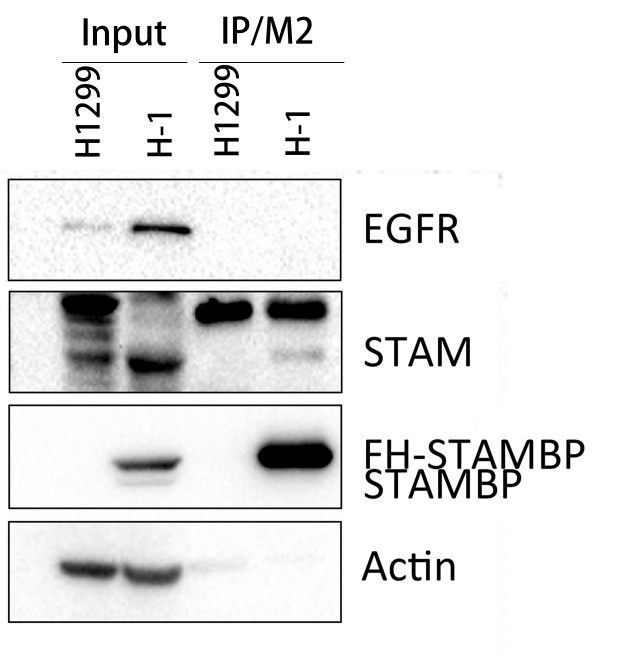
**

**Figure S6 STAMBP can bind to STAM but cannot bind to EGFR in H1299 cells.** Cell extracts were prepared from parental H1299 cells and H-1 cells overexpressing FLAG and HA tagged STAMBP. Co-immunoprecipitation (Co-IP) was performed by FLAG antibody conjugated M2 beads (Sigma, MS, USA). The proteins from input and elutes from Co-IP were detected by the antibodies against EGFR, STAM, STAMBP and β-actin. The ectopic STAMBP expression markedly promotes the stabilization of STAM and EGFR (Lane 1 Vs Lane 2).

**Supplementary figure 7**

**
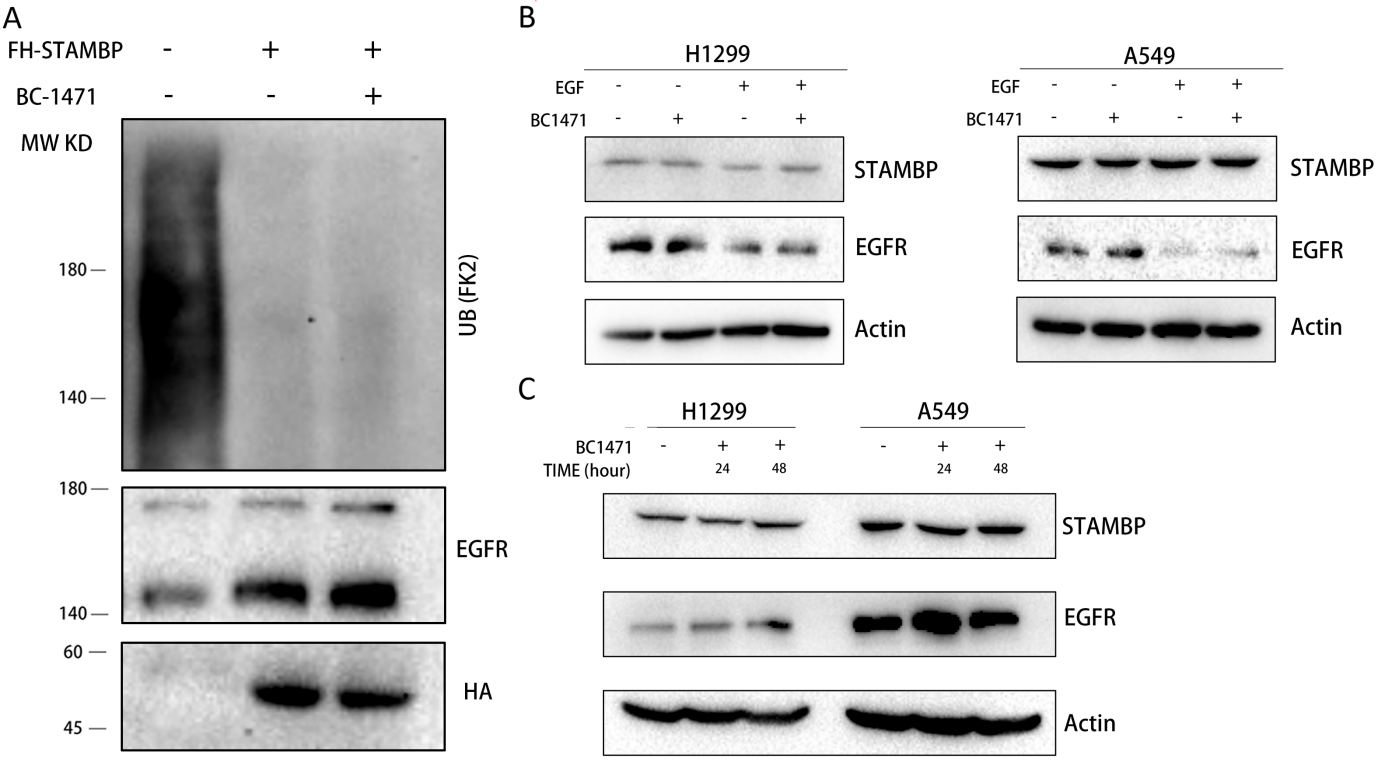
**

**Figure S7 The effects of BC-1471 on STAMBP-mediated deubiquitination and the stability of EGFR.** (A) Purified EGFR complex was incubated with FH-STAMBP without or with 10 µM BC-1471 in a deubiquitination buffer at 37°C for 2 hours. The reaction mixture was resolved by SDS-PAGE for western blot with anti-ubiquitin antibody (upper panel) as well as anti-EGFR and anti-HA antibodies (lower panel). (B) H1299 and A549 cells were left untreated or pretreated with 10 µM BC-1471 2 hours followed by 100 ng/ml of EGF treatment for 15 minutes. (C) H1299 and A549 cells were left untreated or pretreated with 10 µM BC-1471 for 24 and 48 hours. Western blot analysis of the cell extracts was performed with the indicated antibodies (B and C).

**Supplementary figure 8**

**
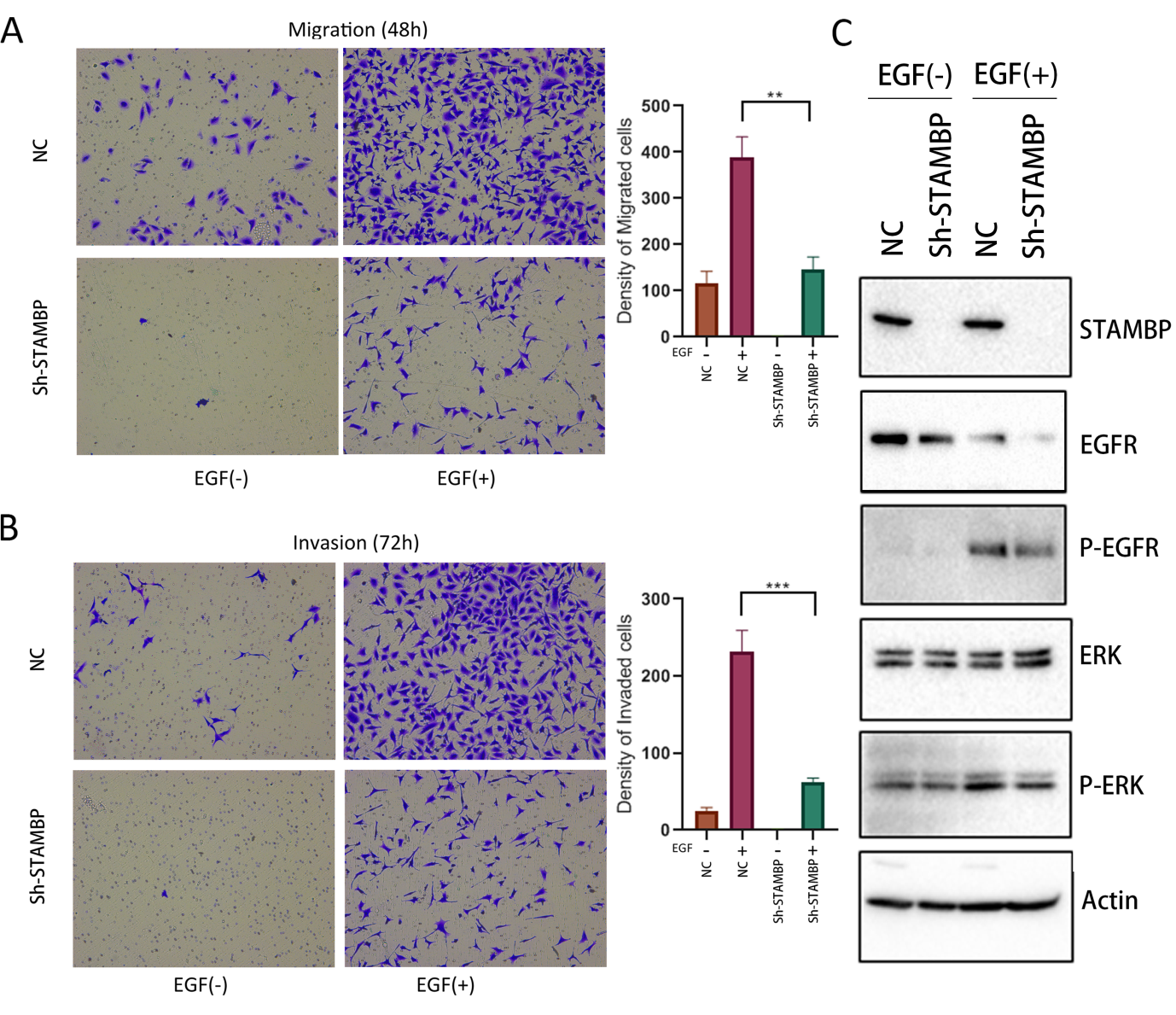
**

**Figure S8 STAMBP stable knockdown inhibits the migration and invasion of A549 cells.** (A and B) A549 cells stably expressing control shRNA and STAMBP shRNA were treated with or without 100 ng/ml of EGF. The number of migrating and invading cells was observed and counted after 48 and 72 hours, [respectively](javascript:;). (C) A549 cells stably expressing control shRNA and STAMBP shRNA were cultured in the presence or absence of 100 ng/ml of EGF for 15 minutes. Western blot analysis of cell extracts with antibody against STAMBP, EGFR, MEK, ERK and the respective phosphorylated proteins. β-actin was used as an internal control. The experiments were repeated twice and representative pictures were shown.

**Supplementary figure 9**

**A**

**
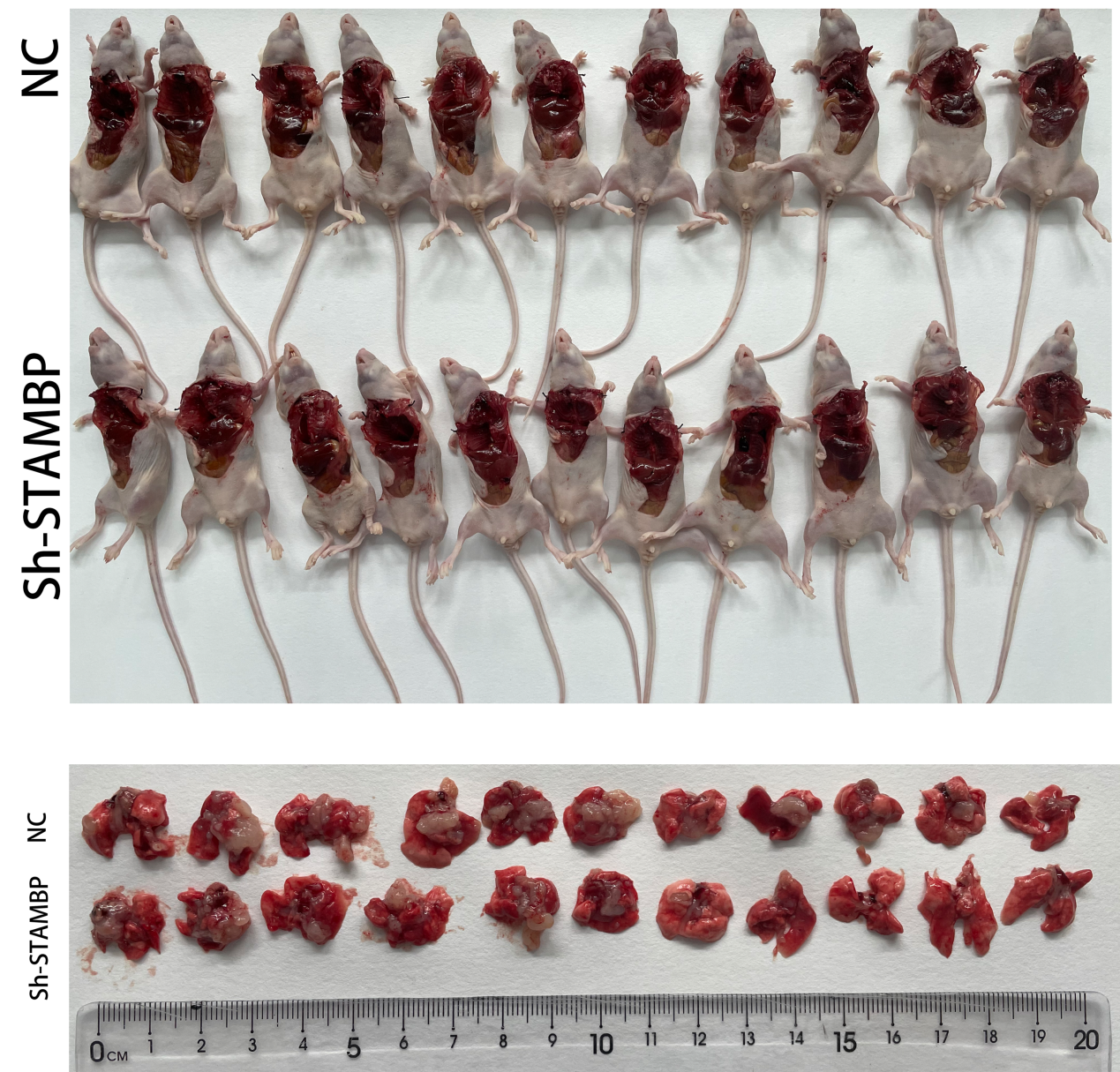
**

**B**


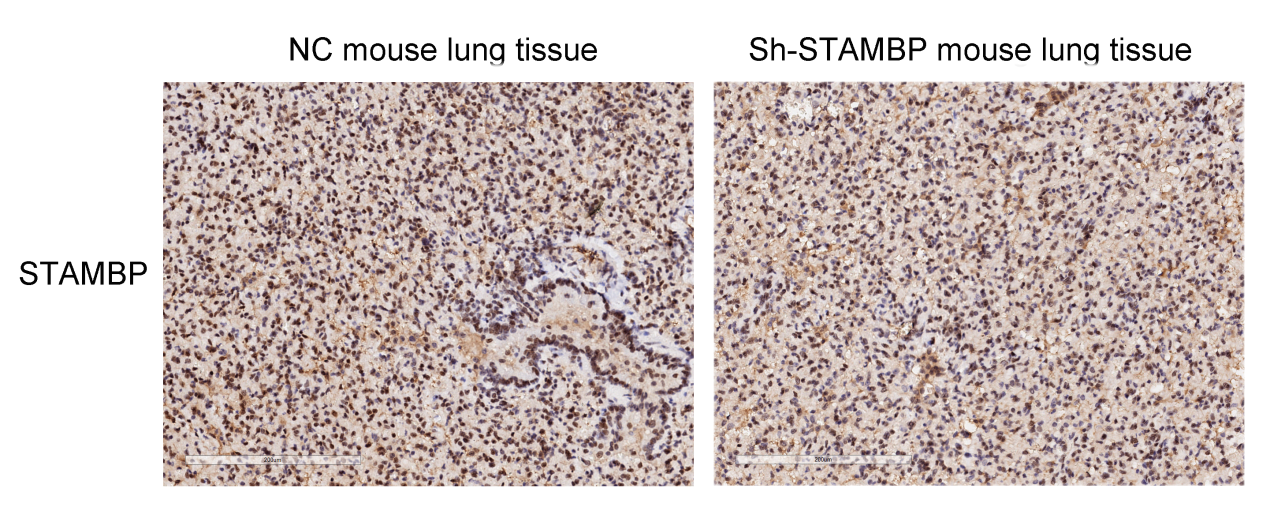


**Figure S9 STAMBP knockdown suppresses tumor growth and metastasis in vivo.** (A) An image of the metastatic tumors on the chest wall and tumors in the lung are shown. (B) STAMBP expression was stained by IHC in the mouse normal lung tissues from the control and STAMBP knockdown groups.
